# Rainwater Charitable Foundation criteria for the neuropathologic diagnosis of progressive supranuclear palsy

**DOI:** 10.1007/s00401-022-02479-4

**Published:** 2022-08-10

**Authors:** Shanu F. Roemer, Lea T. Grinberg, John F. Crary, William W. Seeley, Ann C. McKee, Gabor G. Kovacs, Thomas G. Beach, Charles Duyckaerts, Isidro A. Ferrer, Ellen Gelpi, Edward B. Lee, Tamas Revesz, Charles L. White, Mari Yoshida, Felipe L. Pereira, Kristen Whitney, Nikhil B. Ghayal, Dennis W. Dickson

**Affiliations:** 1 Department of Neuroscience, Mayo Clinic, Jacksonville, FL USA; 2 Department of Neurology, Memory and Aging Center, University of California San Francisco, San Francisco, CA USA; 3 Department of Pathology, University of California, San Francisco, CA USA; 4 Department of Pathology, LIM-22, University of São Paulo Medical School, São Paulo, Brazil; 5 Departments of Pathology, Nash Family Department of Neuroscience, Artificial Intelligence and Human Health, Ronald M. Loeb Center for Alzheimer’s Disease, Icahn School of Medicine at Mount Sinai, Friedman Brain Institute, Neuropathology Brain Bank and Research CoRE, New York, NY USA; 6 Department of Pathology, Boston University School of Medicine, Boston, MA USA; 7 Tanz Centre for Research in Neurodegenerative Disease and Department of Laboratory Medicine and Pathobiology and Department of Medicine, University of Toronto, Toronto, ON Canada; 8 Laboratory Medicine Program and Krembil Brain Institute, University Health Network, Toronto, ON Canada; 9 Banner Sun Health Research Institute, Sun City, AZ USA; 10 Department of Neuropathology, Sorbonne Université, Hôpital de La Pitié-Salpêtrière, Paris, France; 11 Department of Pathology and Experimental Therapeutics, University of Barcelona, Hospitalet de Llobregat, Spain; 12 Department of Neuropathology and Neurochemistry, Medical University of Vienna, Vienna, Austria; 13 Department of Pathology and Laboratory Medicine, Perelman School of Medicine, University of Pennsylvania, Philadelphia, PA USA; 14 Department of Neurodegenerative Diseases, Queen Square Brain Bank for Neurological Disorders, UCL Queen Square Institute of Neurology, University College London, Queen Square, London, UK; 15 Department of Pathology, University of Texas Southwestern Medical Center, Dallas, TX USA; 16 Institute for Medical Science of Aging, Aichi Medical University, Aichi, Japan

**Keywords:** Autopsy cohort, Criteria, Human, Neuropathology, Threads, Oligodendroglia, Phosphorylated tau, Progressive supranuclear palsy (PSP), Neurofibrillary tangles, Tufted astrocytes

## Abstract

Neuropathologic criteria for progressive supranuclear palsy (PSP) proposed by a National Institute of Neurological Disorders and Stroke (NINDS) working group were published in 1994 and based on the presence of neurofibrillary tangles in basal ganglia and brainstem. These criteria did not stipulate detection methods or incorporate glial tau pathology. In this study, a group of 14 expert neuropathologists scored digital slides from 10 brain regions stained with hematoxylin and eosin (H&E) and phosphorylated tau (AT8) immunohistochemistry. The cases included 15 typical and atypical PSP cases and 10 other tauopathies. Blinded to clinical and neuropathological information, raters provided a categorical diagnosis (PSP or not-PSP) based upon provisional criteria that required neurofibrillary tangles or pretangles in two of three regions (substantia nigra, subthalamic nucleus, globus pallidus) and tufted astrocytes in one of two regions (peri-Rolandic cortices, putamen). The criteria showed high sensitivity (0.97) and specificity (0.91), as well as almost perfect inter-rater reliability for diagnosing PSP and differentiating it from other tauopathies (Fleiss kappa 0.826). Most cases (17/25) had 100% agreement across all 14 raters. The Rainwater Charitable Foundation criteria for the neuropathologic diagnosis of PSP feature a simplified diagnostic algorithm based on phosphorylated tau immunohistochemistry and incorporate tufted astrocytes as an essential diagnostic feature.

**Supplementary Information:**

The online version contains supplementary material available at 10.1007/s00401-022-02479-4.

## Introduction

Progressive supranuclear palsy (PSP) is an uncommon neurodegenerative disease with no cure or disease-modifying treatments [7, 11]. PSP causes severe disability and death within 7–9 years after symptomatic onset [4]. PSP is estimated to affect 6 in 100,000 people; however, a higher prevalence has been reported in specific regions, suggesting a role of certain environmental risk factors or genetic variants in increasing the PSP risk [45]. Since PSP was first described in 1963 as a clinicopathological entity [40], significant advances have led to the recognition of PSP as a 4-repeat (4R) tauopathy [10, 13] that often presents as atypical parkinsonism with progressive rigidity, postural instability, early falls and supranuclear gaze palsy [40]. This typical presentation has been referred to as Richardson syndrome [46]. Other clinical presentations are also recognized [25]. PSP is usually a sporadic disease, although mutations in the gene for microtubule-associated protein (*MAPT*) can mimic PSP [21]. Moreover, genetic variants in *MAPT* increase the risk for PSP [24].

The National Institute of Neurological Disorders and Stroke (NINDS) sponsored the first attempt to formulate neuropathologic criteria for PSP over 25 years ago [22]. The NINDS PSP criteria were based upon analysis of cases submitted from participating centers. In the first stage, diagnoses relied on an in-person microscopic review of glass slides provided by participants. This stage was blinded to clinical information. In a second stage, the rater had the opportunity to change diagnosis after disclosure of clinical information [34]. Notably, the criteria were based on a review of glass slides that had not been processed uniformly and included a variety of silver stains and immunohistochemistry. The final NINDS neuropathologic criteria for PSP were based upon the presence and distribution of neurofibrillary tangles and required evaluation of 13 neuroanatomical regions. This extensive sampling scheme may have contributed to the low adoption of these criteria in diagnostic neuropathology settings. Furthermore, inter-rater agreement for typical PSP was moderate and only slight for atypical PSP [34].

Since the NINDS criteria were proposed, immunohistochemistry and availability of commercial antibodies to pathological forms of tau protein, especially phosphorylation-dependent epitopes [6, 18, 30], have facilitated recognition of cardinal neuropathologic features of PSP, including tufted astrocytes [42]. The latter are inconsistently visible with commonly used silver staining methods (e.g., Bielschowsky silver stain). In this report, we describe an effort initiated by neuropathology experts within the Tau Consortium, which is supported by the Rainwater Charitable Foundation, to update neuropathologic criteria for PSP. The proposed criteria incorporate recent advances in tauopathies and a sampling scheme that is applicable in non-specialized neuropathology laboratories. The revised criteria have high sensitivity and specificity and high inter-rater agreement for typical PSP and atypical variants. The criteria are independent of clinical information.

## Materials and methods

### Framework for updating neuropathologic criteria for PSP

The Rainwater Charitable Foundation criteria for the neuropathologic diagnosis of PSP were generated using a multi-step approach: (1) data-driven formulation of provisional neuropathologic criteria, (2) validation of provisional neuropathologic criteria through a blinded inter-rater assessment, and (3) consensus agreement on final neuropathologic criteria after review of the data from the blinded inter-rater study.

Given recent advances in immunohistochemistry for tau since the formulation of the 1994 NINDS PSP criteria, various silver stains and immunohistochemical stains were compared to determine the most sensitive and consistent method for demonstrating PSP pathology. Adjacent 5 µm thick paraffin-embedded tissue sections of PSP cases were stained with the modified Bielschowsky silver stain [47] and the Gallyas-Braak silver stain [9]. Adjacent sections were also processed for immunohistochemistry with two antibodies to phospho-tau—CP13 (pSer202 [23, 29], 1:1000, gift from late Dr. Peter Davies, Feinstein Institute, New York, USA) and AT8 (pSer202/Thr205 1:2500, Thermo Fisher Scientific, USA [6]). While PSP pathologic lesions consist of predominantly 4-repeat tau aggregates [13], 4R tau-specific antibodies are not widely used in diagnostic neuropathology. In contrast, phospho-tau immunohistochemistry is widely used and is recommended in consensus criteria for common neurodegenerative disorders [26]. Each staining method was evaluated by two neuropathologists (DWD, SFR) with a multi head microscope, and there was agreement that immunohistochemistry with the two phospho-tau antibodies was a sensitive and specific method for demonstrating neuronal and glial lesions of PSP. Gallyas-Braak silver stain was nearly as good, but had idiosyncratic case-to-case variability. Immunohistochemistry with a commercially available and widely used monoclonal antibody to phospho-tau, AT8, was chosen for the study.

### Source of material

All the PSP cases and controls had systematic and standardized tissue sampling performed by a single neuropathologist (DWD) to avoid potential bias due to variability in center-specific tissue sampling and processing. The cases were from the Mayo Clinic brain bank, which houses the largest collection of PSP cases diagnosed by a single neuropathologist. The Mayo Clinic brain bank operates with approval from the Biospecimen Committee of the Mayo Clinic Internal Review Board, and autopsy samples are considered exempt from human subject research. All cases were evaluated using conventional methods to analyze common neurodegenerative disorders [38]. This included assessing Alzheimer's type neuropathologic change (ADNC) with Braak neurofibrillary tangle stage [8] and Thal amyloid phase [43] based upon thioflavin S fluorescent microscopy. Other common age-related pathologies, including cerebrovascular pathology, Lewy body pathology, and TDP-43 proteinopathies, were also assessed [3, 14].

### Data-driven formulation of provisional neuropathologic criteria

Neuropathologic data from pathologically confirmed PSP cases in the Mayo Clinic brain bank had semiquantitative lesion data in 21 brain regions [16] that were used to develop provisional criteria (Table 1). Most patients (about 80%) had an antemortem clinical diagnosis of PSP. About 1% of pathologically confirmed PSP were not suspected of having a neurodegenerative disorder, consistent with so-called “incidental PSP” [17]. PSP cases had a median Braak neurofibrillary tangle stage of II (25% quartile: II; 75% quartile: III) and a median Thal amyloid phase of 1 (25% quartile: 0; 75% quartile: 3). The low levels of ADNC were consistent with the fact that only 2% of the patients had a clinical diagnosis of Alzheimer's disease. Comorbid vascular pathology of varying degree, mostly mild, was noted in 23%. Lewy body pathology was detected with α-synuclein immunohistochemistry in 6%; in most cases, it did not exceed the severity seen in incidental Lewy body disease [20]. A few cases had limbic-predominant Lewy body disease [5, 37]. Concurrent TDP-43 proteinopathy was relatively uncommon (5%) and usually more consistent with TDP-43 pathology detected in the elderly (i.e., limbic-predominant age-related TDP-43 encephalopathy; LATE [39]) than with frontotemporal degeneration (FTLD-TDP) [36]. The few cases with FTLD-TDP included about equal frequency of Type B (2%) and Type A (2%), but there were no cases with Type C.

**Table 1:** Mayo clinic PSP brain bank cohort

Demographics of 1,680 PSP cases	
Sex (M:F)	922:758
Non-white	4%
Age at death (median; 25%-, 75%-tile)	70 (75, 81)
Disease duration (median; 25%-, 75%-tile)	5 (7, 9)
Primary clinical diagnosis %	
Progressive supranuclear palsy	81%
Corticobasal degeneration	7%
Parkinson’s disease/dementia	4%
Alzheimer's disease	2%
Dementia with Lewy bodies	1%
Multiple system atrophy	1%
Parkinson-plus	1%
Frontotemporal dementia	1%
Primary progressive aphasia	1%
Amyotrophic lateral sclerosis	< 1%
Normal	< 1%
Vascular	< 1%
Chronic traumatic encephalopathy	< 1%
Other (e.g., CJD, NPH)	1%
Pathology	
Brain weight (median; 25%-, 75%-tile)	1140 (1040, 1240)
Braak NFT Stage (median; 25%-, 75%-tile)	II (II, III)
Thal Amyloid Phase (median; 25%-, 75%-tile)	1 (0, 3)
Co-pathologies (%)	
Vascular-ischemic pathology	23%
Lewy body pathology	6%
TDP-43 pathology	5%

Tau pathology was assessed on sections immunostained with CP13. Lesions were scored on a semiquantitative scale (0 = absent, 1 = mild, 2 = moderate, 3 = severe) for four lesion types (neurofibrillary tangles or pretangles, coiled bodies, tufted astrocytes, threads). Tangle pathology in PSP and throughout this manuscript, often refers to poorly formed neuronal tau lesions (pretangles) or globose neurofibrillary tangles, in contrast to flamed shaped tangles typical of Alzheimer’s disease. Tau positive tufted astrocytes, refers to irregularly deposited tau in astrocytes that often are more prominent in the proximal segments of the astrocytic processes in contrast to other astrocytic tau lesions. Complete tau lesion scores were available on 1565 PSP cases.

Since a recent study on staging of PSP suggested the value in including the calcarine cortex [32], a region not systematically scored for tau pathology, sections from the occipital lobe (Brodmann areas 17, 18) containing primary visual cortex were also processed for phospho-tau immunohistochemistry on a subset of 491 cases. The tau lesion susceptibility data revealed that 99.9% of the PSP cases had neurofibrillary tangles or pretangles in the substantia nigra and subthalamic nucleus, closely followed by the globus pallidus. Tufted astrocytes were frequent in the putamen (99%) and peri-Rolandic cortices (primary and supplementary motor cortex, i.e., Brodmann area 4, 6) (96%). Although tufted astrocytes were also found in the subthalamic nucleus (76%), they were less frequent than in the putamen and peri-Rolandic cortices. Tufted astrocytes were also less frequent in the globus pallidus (42%) and substantia nigra (43%) (Table 2).

**Table 2:** Frequency of Tau lesions in Mayo Clinic PSP brain bank

Region/lesion *N* = 1565	Neurofibrillary tangles or pretangles		Coiled bodies		Tufted astrocytes		Threads	
	Absent	Present	Absent	Present	Absent	Present	Absent	Present
Peri-Rolandic cortices	1% (20/1545)	99%	3.4% (53/1543)	96%	4% (56/1545)	96%	9% (137/1543)	91%
Putamen	0.1% (2/1560)	99.9%	2.2% (34/1562)	98%	1% (12/1562)	99%	9% (139/1561)	91%
Globus pallidus	0.4% (7/1555)	99.6%	5.4% (84/1554)	95%	42% (652/1554)	58%	4% (65/1554)	96%
Subthalamic nucleus	0.1% (2/1553)	99.9%	4.0% (62/1553)	96%	24% (374/1553)	76%	1% (8/1551)	99%
Substantia nigra	0.1% (1/1556)	99.9%	9.0% (140/1556)	91%	43% (663/1556)	57%	0.1% (2/1555)	99.9%

Absence versus presence of individual lesions in criteria specific regions. NA scores were excluded from calculations

Next, we generated a tau burden dataset for each region and each tau lesion type (presence/absence), analyzing over 120,000 semiquantitative scores of tau lesion severity. We also created a semiquantitative tau lesion susceptibility map [median scores 0–3 (Table 3)]. From these maps, we selected five regions to include in the provisional PSP criteria. Regions were selected based on the burden of tau pathology in PSP. Particular emphasis was given to selecting brain regions that would overlap as much as possible with the widely used NIA-AA sampling scheme [38] (Table 4). The peri-Rolandic cortices (sampling Brodmann areas 1, 3, 4, 6) are the only region in the updated PSP criteria not included in the NIA-AA recommended sampling scheme. The rationale for including the peri-Rolandic cortices rather than the more commonly sampled middle frontal cortices was based on the data analysis of neuronal and glial lesions in these two regions and the need to provide criteria that unequivocally captures the archetypical PSP lesions.

**Table 3:** Median tau scores from the Mayo Clinic brain bank

Region (*N* = 1565)	Neurofibrillary tangles or pretangles	Tufted astrocytes	Oligodendroglia coiled bodies	Threads
Frontal cortex	2	2	2	1
Peri-Rolandic cortices	2	3	2	2
Putamen	2	3	2	1
Globus pallidus	2	1	2	2
Subthalamic nucleus	3	2	2	3
Substantia nigra	2	1	1	2
Cerebellar dentate nucleus	2	0	1	2
Occipital cortex	0	1	0	0

Semiquantitative lesion scores (0 = none, 1 = mild, 2 = moderate, 3 = marked)

**Table 4:** Neuroanatomical regions in Rainwater Charitable Foundation criteria for the neuropathologic diagnosis of PSP compared with NIA-AA recommendations

	NIA-AD recommendations	Mayo Clinic brain bank	Inter-rater study	Final rainwater charitable foundation criteria
Cerebral cortex	Middle frontal	Superior frontal	Middle frontal	
	Superior/middle temporal	Inferior temporal		
		Motor cortex	Peri-Rolandic cortices	Peri-Rolandic cortices
	Inferior parietal			
	Occipital		Occipital	
	Cingulate			
Limbic	Hippocampus		Hippocampus	
	Amygdala		Amygdala	
	Basal nucleus	Basal nucleus		
Basal ganglia	Globus pallidus	Globus pallidus	Globus pallidus	Globus pallidus
	Putamen	Putamen	Putamen	Putamen
Diencephalon		Hypothalamus		
	Thalamus	Thalamus		
	Subthalamic nucleus	Subthalamic nucleus	Subthalamic nucleus	Subthalamic nucleus
Midbrain	Substantia nigra	Substantia nigra	Substantia nigra	Substantia nigra
		Red nucleus		
		Oculomotor nucleus		
		Tectum		
Pons	Locus ceruleus	Locus ceruleus		
		Tegmentum		
		Pontine base		
Medulla	Dorsal motor nucleus			
		Tegmentum		
		Inferior olive		
Cerebellum	Cerebellum			
		Dentate nucleus	Dentate nucleus	
		White matter		

## Validation of provisional neuropathologic criteria in a blinded inter-rater assessment

### Case selection and tissue preparation for the inter-rater assessment

A total of 25 cases were selected to validate the provisional neuropathologic criteria for PSP, including 10 typical PSP, 5 atypical PSP, and 10 cases with another primary tauopathy. All cases were free of Lewy body pathology, TDP-43 proteinopathy, and significant vascular pathologies. Given the focus on determining critical features that could differentiate PSP from other tauopathies, most cases also lacked ADNC or other age-related tau pathologies. The demographics of the inter-rater study cases are summarized in Table 5.

**Table 5:** Demographics and pathology of PSP and other tauopathy cases in the study cohort

Group	Sex (F:M)	Age at death (years)	Duration (years)	Braak stage	Thal phase	Brain weight (grams)
PSP (*N* = 15)	1:2	68.3 (57–87)	6.2 (2–10)	1.1	0.7	1170
Other tauopathies (*N* = 10)	1:2	63.5 (41–85)	5.8 (0–10)	3	1.1	1150

*F* female, *M* male, Braak stage = Alzheimer neurofibrillary tangle stage, Thal phase = amyloid plaque phase. Brain weight is whole brain weight calculated from doubling the weight of the hemibrain

Inclusion criteria for the ten typical PSP required an antemortem clinical diagnosis of PSP–Richardson syndrome [46]. The cases represented a range of neuropathologic severity of PSP-tau pathology; none had more than low ADNC. Similar to typical PSP, atypical PSP cases had to meet NINDS neuropathologic criteria for PSP [15]. Atypical PSP cases had features such as a predominant cortical or hindbrain tau pathology, pallidonigroluysial atrophy or histopathologic features resembling globular glial tauopathy (GGT) type II [1]. The ten other primary tauopathy cases included diffuse argyrophilic grain disease (DAGD) the equivalent of Saito stage 3 [19], chronic traumatic encephalopathy (CTE), corticobasal degeneration (CBD), and globular glial tauopathy (GGT types I and III), Pick's disease and FTLD-tau due to *MAPT* mutation (N279K). All cases had low or no ADNC. Atypical PSP and other primary tauopathies were age- and sex-matched to typical PSP.

### Inter-rater study design and data capturing

Inter-rater assessment of the 25 cases was based upon viewing digitized images of paraffin-embedded tissue sections cut a 5 µm-thickness. The eight slides included ten anatomical regions of interest. Sections were stained with hematoxylin and eosin, and adjacent sections were immunostained with a commercially available monoclonal antibody to phosphorylated tau (AT8; pSer202/Thr205 1:2500, Thermo Fisher Scientific, USA) and counterstained with hematoxylin.

**Fig. 1:**
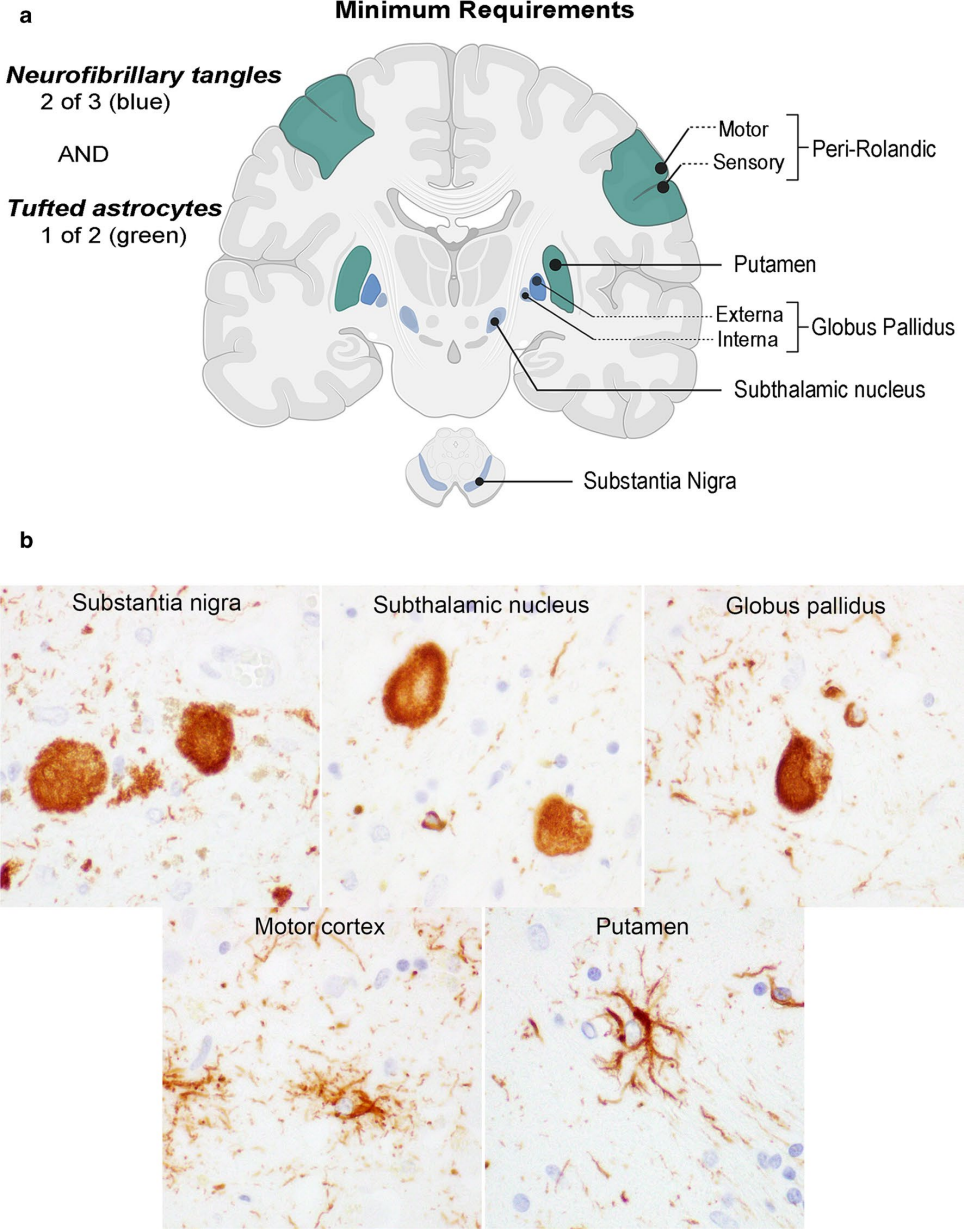
**A** schematic representation of tau lesions included in the Rainwater Charitable Foundation criteria for the neuropathologic diagnosis of PSP (green shading = tufted astrocytes, blue shading = tangles or pretangles). To fulfill a PSP diagnosis, tufted astrocytes need to be present in a minimum of one of two regions (green shading) AND neurofibrillary tangles need to be present in a minimum of two of three regions (blue shading) **B** based upon tau immunohistochemistry. Scale bar = 20 µm. All images were captured at 60 × magnification on an Olympus BX41 microscope, using digital the camera DP22

H&E- and AT8-stained sections were digitized at 40 × magnification using an Aperio scanning system (Leica Biosystems, Buffalo Grove, IL, USA). Digitized images were labelled with the anatomical regions of interest in Aperio ImageScope software (Leica Biosystems, Buffalo Grove, IL, USA). Cases were numbered (1 through 25) based on the acquisition date. All raters received access to the same set of digital images. Each rater received access to a secured Aperio server hosted at the Mount Sinai School of Medicine, New York, NY, or they received a hard drive holding the set of images and a free version of Aperio ImageScope software (Leica Biosystems, Buffalo Grove, IL, USA). Raters were blinded to demographics, as well as clinical and neuropathological information. They were instructed to score the severity of tau lesions using a four-point semiquantitative scale (0–3) according to their personal preferences. Raters were also provided an instructional booklet containing a brief description of the typical findings for each tauopathy, and a table showing median regional tau lesion scores from the Mayo Clinic brain bank (Supplementary Fig. 1, online resource) and provisional neuropathologic criteria for PSP (Fig. 1a). The scoring datasheet for the raters was developed using a digital data capture platform (Qualtrics XM, Provo UT, USA) hosted by the University of California San Francisco to allow each rater to input their tau lesion-specific semiquantitative scores for each brain region from a drop-down menu. Finally, each rater was required to provide a categorical diagnosis of PSP or not-PSP based upon the minimal criteria summarized in Table 6, which required neurofibrillary tangles or pretangles (Fig. 1b, top row) in two of three regions (substantia nigra, subthalamic nucleus, globus pallidus) and tufted astrocytes (Fig. 1b, bottom row) in one of two regions (peri-Rolandic cortices, putamen). Any additional comments were registered in an accompanying text box.

**Table 6:** Minimal criteria for neuropathologic diagnosis of PSP

Regions	Tau lesions		Minimum Requirements
	Neurofibrillary tangles or pretangles	Tufted astrocytes	
Globus pallidus	≥ 1 +		Two of three regions
Subthalamic nucleus	≥ 1 +		
Substantia nigra	≥ 1 +		
AND			
Peri-Rolandic cortices		≥ 1 +	One of two regions
Putamen		≥ 1 +	

Minimum required lesions and regions to be evaluated for diagnosis of PSP: neurofibrillary tangles or pretangles in a minimum of two of three regions (globus pallidus, subthalamic nucleus, substantia nigra) and tufted astrocytes in a minimum of one of two regions (peri-Rolandic cortices, putamen). ≥ 1 + requires at least a mild frequency of lesions, intended to indicate more than one to two lesions within the region-of-interest

### Statistical methods

The inter-rater assessment used a blinded study design. The two data analysts (F.L.P., N.B.G.) were not involved in rater assessments. The raw data from inter-rater assessments were downloaded from Qualtrics XM, including categorical data (diagnosis of PSP or not-PSP). Regional tau lesion scores from all 14 raters using their personal preference were tested using the Krippendorff’s alpha [48] (Supplementary Fig. 2, online resource). Sensitivity and specificity were calculated using the Mayo Clinic brain bank diagnoses as a gold standard. The percentage of inter-rater agreement was summarized based on the categorical diagnoses provided by each rater (Supplementary Fig. 3, online resource). For stringency, all calculated inter-rater kappa coefficients were standard fixed Fleiss' Kappa values [12]. Kappa was calculated for all samples and was independent of group (i.e., typical PSP cases, atypical PSP cases, and other tauopathies). Kappa coefficients were grouped as almost perfect agreement (0.81–1.0), substantial agreement (0.61–0.80), moderate agreement (0.41–0.60), fair agreement (0.21–0.40) or slight agreement (0.01–0.20) [33]. Every rater was evaluated using the mean + 1 standard deviation (SD) to determine outliers who may have skewed the results. The second set of analyses used tau pathology lesion scores provided by the raters in five areas independent of the diagnosis. Finally, the burden of tufted astrocytes was evaluated using scores provided by the raters for each brain region. A comparison of PSP or not-PSP for each region was conducted with using Mann–Whitney Rank Sum test. Statistical analyses were performed with R-software (Version 4.1.1). Sensitivity and specificity were calculated using "caret" library. Krippendorff’s alpha using the kripp.boot” with a bootstrap statistic of 20,000, for 25% and 75% percentiles and Fleiss kappa tests were performed using “DescTools” library. All *p* values were two-sided, and a *p* value of *p* < 0.05 was considered statistically significant. All values reported as 0 were denoted as *p* < 0.0001.

## Results

### Specificity, sensitivity, and inter-rater agreement of the categorical criteria

When classifying cases based on the categorical diagnosis (PSP or not-PSP), the 14 raters had high sensitivity (0.94, 95% CI 0.89–0.96) and specificity (0.96, 95% CI 0.90–0.98) for the diagnosis of PSP when compared to the Mayo Clinic brain bank diagnoses. Inter-rater reliability showed almost perfect agreement for neuropathological diagnoses for all 25 cases (Fleiss Kappa 0.837, 95% CI 0.796–0.878, *p* < 0.0001). No outlier was identified among the reviewers using the mean + 1 SD to evaluate rater bias.

The sensitivity varied between typical PSP (0.96, 95% CI 0.91–0.98) and atypical PSP (0.89, 95% CI 0.78–0.94). Accordingly, the agreement was almost perfect (Fleiss Kappa 0.854, 95% CI 0.808–0.900, *p* < 0.0001) when evaluating typical PSP against not-PSP, whereas the inter-rater agreement dropped to substantial agreement when comparing atypical PSP vs. not-PSP (Fleiss Kappa 0.796, 95% CI 0.743–0.849, *p* < 0.0001). The lower inter-rater reliability for atypical PSP was driven by a case with GGT type II (percentage agreement 43%). The other four atypical PSP cases had a 100 percent inter-rater agreement. Atypical PSP and GGT type II share neuropathological features, and there is no current consensus on whether GGT type II is a variant of PSP or a unique subtype of tauopathy [1]. We re-evaluated the atypical PSP cases removing the GGT type II case. This led to an increase in sensitivity (0.97, 95% CI 0.93–0.98) and retained high specificity (0.96, 95% CI 0.91–0.98) with almost perfect inter-rater agreement (Fleiss Kappa 0.874, 95% CI 0.833–0.916, *p* < 0.0001).

## Specificity, sensitivity, and inter-rater agreement of the provisional criteria when considering rater tau scores

Acknowledging that all participating raters were experts in PSP neuropathology and had access to 12 digitized images of 10 brain areas (the criteria include only five areas), we wanted to evaluate whether expertise would bias the final diagnosis. To evaluate this hypothesis, we reassigned the diagnosis to each case based solely on unsupervised scoring for each rater based upon five areas (peri-Rolandic cortices, putamen, globus pallidus, subthalamic nucleus, and substantia nigra).

When considering any level of positivity (1–3) as a positive score, the specificity dropped (0.84, 95% CI 0.76–0.89), while the sensitivity remained high (0.94, 95% CI 0.90–0.97) compared to the rates obtained based upon categorical diagnoses. Expectedly, inter-rater agreement dropped, although it continued to show a substantial agreement (Fleiss Kappa 0.667, 95% CI 0.626–0.708, *p* < 0.0001). Sensitivity remained high in differentiating typical PSP (0.95, 95% CI 0.89–0.97) and atypical PSP (0.92, 95% CI 0.84–0.97). When typical PSP and atypical PSP categories were evaluated and compared to not-PSP, the inter-rater reliability was substantial for typical PSP (Fleiss Kappa 0.657, 95% CI 0.611–0.703, *p* < 0.0001) and moderate for atypical PSP (Fleiss Kappa 0.576, 95% CI 0.523–0.629, *p* < 0.0001). Removing the GGT type II case led to improved sensitivity (0.96, 95% CI 0.93–0.99), specificity (0.84, 95% CI 0.76–0.89) and inter-rater agreement (Fleiss Kappa 0.697, 95% CI 0.655–0.739, *p* < 0.0001).

Analysis of the tau data confirmed an increased number of false-positive diagnoses for PSP compared to the “categorical diagnoses.” The increase was driven by the assessment of tufted astrocytes. A few raters interpreted rare astroglial tau inclusions in the putamen or peri-Rolandic cortices as sufficient to fulfill the criteria for tufted astrocytes. The data were analyzed to determine if other combinations of scores in the cardinal regions could improve the diagnostic accuracy against the Mayo Clinic brain bank diagnosis. None of the various combinations evaluated gave a better positive predictive value. The second-best scheme was to require a tufted astrocyte score of more than 1 (instead of ≤ 1) in either putamen or peri-Rolandic cortices (sensitivity: 0.84, 95% CI 0.79–0.89; specificity: 0.92; 95% CI 0.86–0.96; Kappa: 0.73, 95% CI 0.69–0.78).

### Tufted astrocytes have diagnostic value in PSP

For lack of a reliable detection method, tufted astrocyte pathology was not included in the 1994 PSP criteria. Our observations showed that PSP cases had a significantly higher burden of tufted astrocytes than other disorders for each region examined, except for the cerebellar dentate nucleus, where tufted astrocytes were minimal or absent in most PSP cases (Table 7). Other regions with few or no tufted astrocytes include the hippocampus and subthalamic nucleus. In PSP, tufted astrocytes were sparse in globus pallidus, substantia nigra, and occipital cortex (Table 2). Of interest is that tufted astrocytes were a consistent feature of PSP, including a case of “incidental PSP” in a patient with a disease duration of fewer than three years and mild tau pathology, including sparse tufted astrocytes in the putamen.

**Table 7:** Comparison of tufted astrocyte scores in PSP and other tauopathies

Anatomical region	Tufted astrocytes
Midfrontal cortex	*P* < 0.001
Peri-Rolandic cortices	*P* < 0.001
Occipital cortex	*P* = 0.003
Hippocampus	*P* = 0.003
Amygdala	*P* < 0.001
Globus pallidus	*P* = 0.002
Putamen	*P* < 0.001
Subthalamic nucleus	*P* < 0.001
Substantia nigra	*P* = 0.003
Dentate nucleus	n.s

Mann–Whitney Rank sum test. All *p* values are double-sided

## Discussion

The 1994 NINDS neuropathologic criteria for PSP, based primarily on the distribution of neurofibrillary tangles with silver staining methods, formed the basis for the neuropathologic diagnosis of PSP until now. At the time, an inter-rater assessment study showed that these criteria had only moderate inter-rater reliability for typical PSP and only slight inter-rater reliability for atypical PSP [34]. Additional unblinded information was necessary to increase the inter-rater agreement to substantial [34]. We hypothesized that using tau immunohistochemistry and defining cardinal regions and lesion types using a data-driven approach might improve inter-rater reliability for neuropathologic diagnosis of PSP. The regions and lesion types included in the provisional criteria were selected based on data of tau lesion scores and their neuroanatomical distribution in 1,565 PSP cases from the Mayo Clinic brain bank.

The focus of the current study was to identify concise and reproducible means to evaluate and diagnose PSP in both expert and non-expert settings. To determine the reliability and validity of this approach, we conducted a blinded inter-rater study. The provisional criteria utilized a simplified diagnostic algorithm based upon the presence of neurofibrillary tangles or pretangles in three cardinal nuclei (substantia nigra, subthalamic nucleus, and globus pallidus) and tufted astrocytes in two regions (peri-Rolandic cortices or putamen) (Table 6). The provisional criteria showed very high sensitivity, high specificity, and substantial inter-rater reliability for diagnosing both PSP and atypical PSP.

The PSP regions of interest may not apply to other more common neurodegenerative disorders, such as Alzheimer's and Lewy body disease. In particular, peri-Rolandic cortices are not commonly sampled in neuropathology laboratories focused on diagnosing common age-related pathologies [2, 38] (Table 4). Analysis of the aggregate data was critical in identifying diagnostically important histologic features in both relatively pure PSP and atypical PSP and diagnostically significant brain regions. Peri-Rolandic cortices provided the best value of the cortical regions tested and removing this area affected both sensitivity and specificity of the criteria.

A novel feature of the updated PSP criteria is the diagnostic importance of tufted astrocytes. In contrast, the 1994 NINDS criteria were focused only on neuronal lesions [22]. The value of astrocytic pathology is that it permits recognition of even early or mild PSP cases (“incidental PSP”). The current study did not address the diagnostic value of oligodendroglial coiled bodies. While coiled bodies are a characteristic feature of PSP, they lack specificity and are found in other neurodegenerative tauopathies such as AGD, CBD, and GGT [28]. Notably, oligodendroglial lesions were an exclusion criterion for PSP in the 1994 NINDS PSP criteria [22].

Comparing the Mayo Clinic PSP-tau scores and the inter-rater unsupervised tau scores revealed that the subthalamic nucleus and the globus pallidus were key drivers of a neuropathologic diagnosis of PSP, in contrast to the substantia nigra. The lentiform nucleus is included in widely used recommended sampling schemes [2, 38] and is readily identified in brain sections, but the same cannot be said for the subthalamic nucleus, which can be difficult to detect macroscopically depending upon the plane of section and on the thickness of the brain section. In this case, the criteria require neurofibrillary tangles or pretangles in both cardinal regions (i.e., globus pallidus and substantia nigra). The midbrain is included in recommended sampling schemes for age-related neurodegenerative disorders. Still, the focus on the substantia nigra in the updated PSP criteria needs to consider the fact that the involvement of this region in cases with concurrent Alzheimer's disease may be problematic since the substantia nigra is often affected by neurofibrillary pathology in aging and AD [35, 44], albeit glial tau pathology in the midbrain section is not a feature of ADNC. While not included in the minimal essential regions of the updated PSP criteria, other regions in the midbrain section may offer more diagnostic information for PSP in such cases since the red nucleus and the midbrain tectum are rarely affected in aging and AD. For similar reasons, the peri-Rolandic cortices, particularly Brodmann area 4, were prioritized over the middle frontal cortex, although the latter showed only mildly lower median scores in neurofibrillary tangles or pretangles and tufted astrocytes. The updated PSP criteria do not specifically address the value of these regions in diagnosis and differential diagnosis.

The updated neuropathologic criteria for PSP need to be validated in a non-expert setting, where sampling and staining methods may depart from the practice in a specialized center, especially concerning the value of assessing subthalamic nucleus and peri-Rolandic cortices. Given the diagnostic importance that the updated PSP criteria place on tufted astrocytes, it is uncertain if nonexperts can recognize characteristic tau-positive glial lesions (Supplementary Fig. 4, online resource) in PSP and their ability to distinguish them from astrocytic lesions in other tauopathies (Supplementary Fig. 5, online resource), including common age-related tauopathies such as AGD [27] and ARTAG [31]. Furthermore, GGT type II was difficult to differentiate from PSP even among a panel of experts, a finding corroborated by ultrastructural studies of tau filament folds [41], suggesting that this may be particularly difficult in exceptional cases. Nevertheless, the results of the current study highlight several advantages of the updated neuropathologic criteria for PSP over the NINDS PSP criteria. Not only did the updated criteria show better inter-rater agreement, but they also require less extensive sampling. Moreover, the regions of interest were chosen to overlap with current sampling schemes for common age-related disorders nucleus, except the inclusion of the peri-Rolandic cortices. In addition, the neuropathologic diagnosis of PSP in the 1994 NINDS criteria incorporated clinical information. In contrast, the updated PSP criteria are independent of clinical information.

## Recommendations

### The rainwater charitable foundation criteria for the neuropathologic diagnosis of PSP

Based on objective results of the inter-rater study and discussions of the PSP Criteria Study Group, the following features are recommended for a neuropathologic diagnosis of PSP:

1. Neurofibrillary tangles or pretangles, at least mild in frequency, in two or more of the following regions: globus pallidus, subthalamic nucleus and substantia nigra
2. Tufted astrocytes, at least mild in frequency, in either peri-Rolandic cortices or putamen

“At least mild in frequency” is intended to indicate more than 2 lesions within the region-of-interest. These criteria do not preclude evaluation of additional brain regions that may provide insight into other neurodegenerative processes, including common age-related conditions or in disease staging.

## Supplementary Information

Below is the link to the electronic supplementary material.

Supplementary file1 (PDF 2761 kb)

Supplementary file2 (DOCX 10272 kb)
