# Supplementary material for: Rainwater Charitable Foundation criteria for the neuropathologic diagnosis of progressive supranuclear palsy
Source: Acta Neuropathol. 2022 Aug 10;144(4):603–14. doi: 10.1007/s00401-022-02479-4 (PMC9468104; doi:10.1007/s00401-022-02479-4)
Supplement: Supplementary file 1 — Supplementary file1 (PDF 2761 kb) [file 401_2022_2479_MOESM1_ESM.pdf]

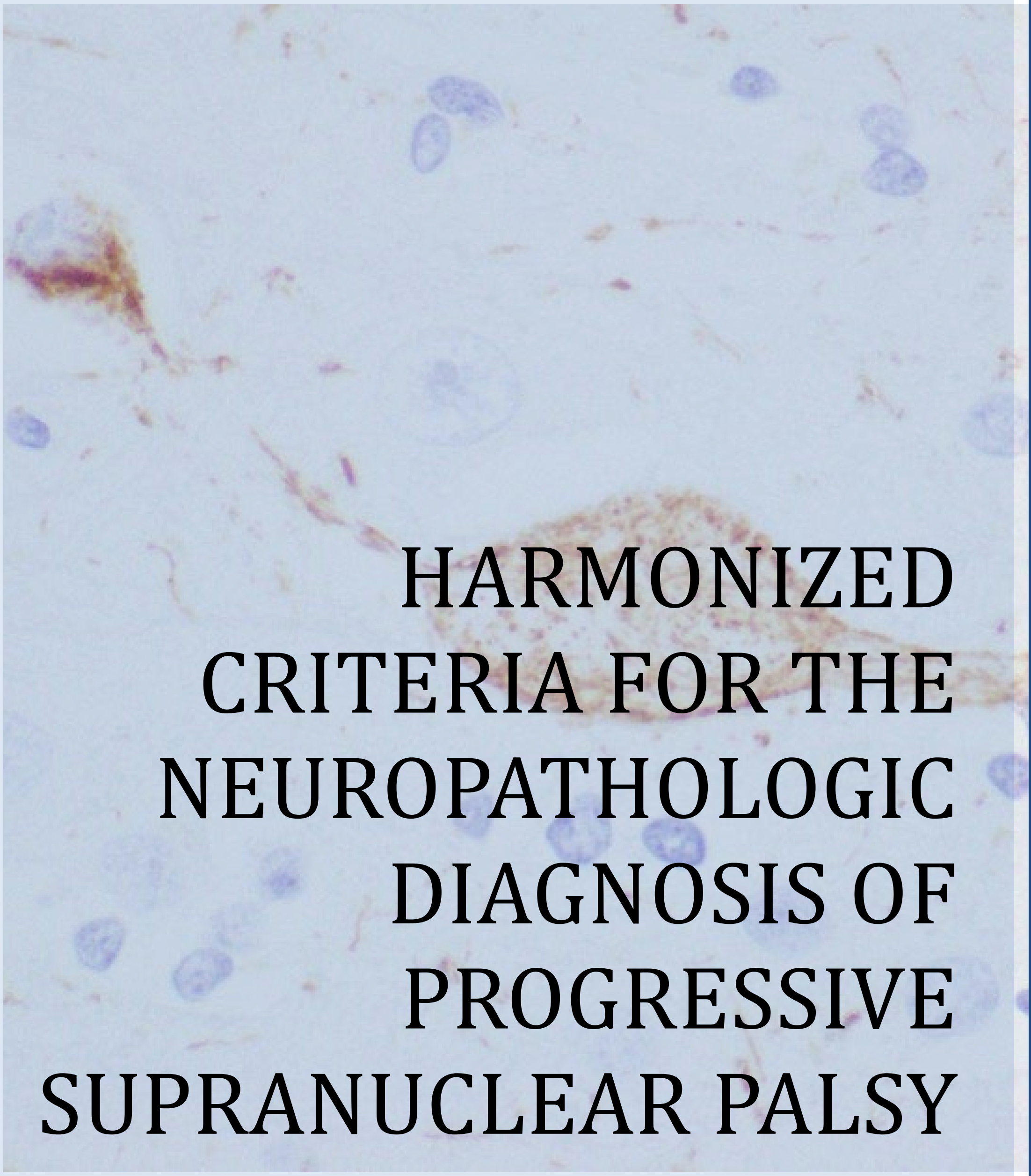

HARMONIZED CRITERIA FOR THE NEUROPATHOLOGIC DIAGNOSIS OF PROGRESSIVE SUPRANUCLEAR PALSY

A practical approach

Shanu F. Roemer. Lea T. Grinberg.
John F. Crary. Dennis W. Dickson

Supported by
The Rainwater
Charitable
Foundation,
&
The National Institute
of Health

©Mayo Clinic and College of Medicine, 2021
This work is subject to copyright.
All rights are reserved whether whole or part of the material is concerned. This specifically concerns the rights of translation, printing, use of illustrations, recitations, broadcasting, reproduction on digital images, microfilm and storage in databanks. Duplication of this publication or parts thereof is only permitted under the provisions of authors and Mayo Clinic and College of Medicine.

Contents

v.	Prologue	p. 3
vi.	How to use the slide images	p. 4
vii.	How to use the booklet	p. 5
1.0.	Selected anatomical regions	p. 6
1.1.	Macroscopic findings in typical PSP	p. 7
1.2.	Cardinal brain nuclei in typical PSP	p. 7
1.3	Tau pathology in PSP	p. 7
1.4.	Provisional neuropathologic diagnostic criteria of typical PSP	p. 8
2.0.	Typical PSP	p. 8 - 11
3.0.	Atypical PSP	p. 11
3.1.	Cortical variant	p. 11
3.2.	PSP with globular inclusions	p. 12
3.3.	Hindbrain predominant	p. 12
3.3.	PSP-PNLA	p. 12-13
3.5.	Differential diagnostic considerations	p. 12
4.0.	Other Tauopathies	p. 13-18
4.1.	Globular Glial tauopathy type 1	p. 13
4.2.	Globular Glial Tauopathy type 2	p. 13
4.3.	Globular Glial Tauopathy type 3	p. 13 - 14
4.4.	Corticobasal degeneration	p. 14 - 15
4.5.	Argyrophilic grain disease	p. 15 - 16
4.6.	Pick's disease	p. 16 - 17
4.7.	Chronic Traumatic Encephalopathy	p. 17
4.8.	Tauopathies with known MAPT mutation	p. 17-18
viii.	Photo Atlas	p. 19 - 42
ix.	Block key	p. 43
x.	Required PSP lesions	p. 44
xi.	Scoring sheet	p.45
xi.	Summary of provisional PSP criteria	p. 46
xii.	Contact information	p. 47

Prologue

Progressive supranuclear palsy (PSP) is 4R tauopathy and the most frequent disease in the spectrum of primary tauopathies. Clinically it is characterized by heterogeneity of the most predominant features such as severe postural instability and supranuclear gaze palsy (Richard syndrome), pure akinesia and gait failure (PAGF), rigidity and dopamine responsiveness (PSP-P) or marked cerebellar ataxia (PSP-C). The average disease duration is 7 years, but rapidly progressive forms (<3 years) or long disease duration (>15 years) exist.

There is high concordance between the antemortem clinical diagnosis and autopsy-confirmed PSP, as previously published by Litvan *et al.*, and confirmed in the Eloise Troxel Memorial PSP brain bank (ca. 80%). Despite various clinical presentations, the distribution of neuropathological features is relatively stereotypic, but there is heterogeneity with respect to the degree of neuronal loss, glial tau lesions and neurofibrillary pathology depending upon the clinical presentation.

The published neuropathologic criteria for PSP by Hauw *et al.* relies on the distribution of neurofibrillary tangles in cardinal nuclei for a diagnosis. Meticulous and systematic assessment of glial tau lesions in the Eloise Troxel Brain bank; however, should engage the discussion whether the extent of glial tau lesions or the morphology may also be considered in the pending diagnostic criteria revision (Tables 2 & 3).

We have chosen cases to represent relatively pure PSP, with minimal co-morbid pathology for typical cases. Patients all have an antemortem clinical diagnosis of PSP. Atypical PSP and other tauopathies may contain other pathologic processes, especially for older patients. The priority of 'pure' PSP was given over consideration of demographic diversity. Unfortunately, the autopsy cohort has male predominance and is predominantly Caucasian (96%). We anticipate the selection of atypical PSP cases will spark a lively debate upon study completion. For these cases, a diagnosis of PSP with or without subtyping of its variant, suffice for the subsequent grouping of cases.

Table 1.

Group	Sex (F:M)	Race	Age in years	Duration in years	Braak	Thal	Brain wt
PSP (N=15)	1:2	Caucasian	68.3 (57-87)	6.2 (2-10)	1.1	0.7	1170 g
Other tauopathies (N=10)	1:2	Caucasian	63.5 (41-85)	5.8 (0-10)	3	1.1	1150 g

Average Braak NFT stage, Thal amyloid phase (based upon thioflavin S fluorescent microscopy), and brain weight, duration and age (range).

Litvan I, Hauw JJ, Bartko JJ, et al. Validity and reliability of the preliminary NINDS neuropathologic criteria for progressive supranuclear palsy and related disorders. *J Neuropathol Exp Neurol* 1996;55:97-105.

Hauw JJ, Daniel SE, Dickson D, et al. Preliminary NINDS neuropathologic criteria for Steele-Richardson-Olszewski syndrome (progressive supranuclear palsy). *Neurology* 1994;44:2015-2019.

How to use the slide images

You have received a secured access to the Mt. Sinai Aperio server. Please use the supplementary instructions you have received in your email on how best to use Aperio if you are not familiar with the software. For issues related to problems with the use of the server, please see the contact information on page 49.

Each tauopathy on the hard-drive contains H&E and corresponding AT8 for each individual case. A block key is provided on page 43.

Generally, H&E's show a more intensified hue compared to viewing the slides under the microscope. Hue, saturation and brightness can be modified to your liking under the button 'Image' button and choosing 'Adjustment' in the drop-down menu. Anatomical regions can be better differentiated using 'adjustment' change the color curve.

A benefit of the ImageScope compared to microscopic images is that the images can easily be zoomed in and out, right clicking the mouse and using the scroll button on your mouse or keyboard, in order to give a detailed versus full slide over view.

Additionally, annotations can be added and saved for future questions to be addressed, using the arrow, rectangle, ruler, counting tool etc.

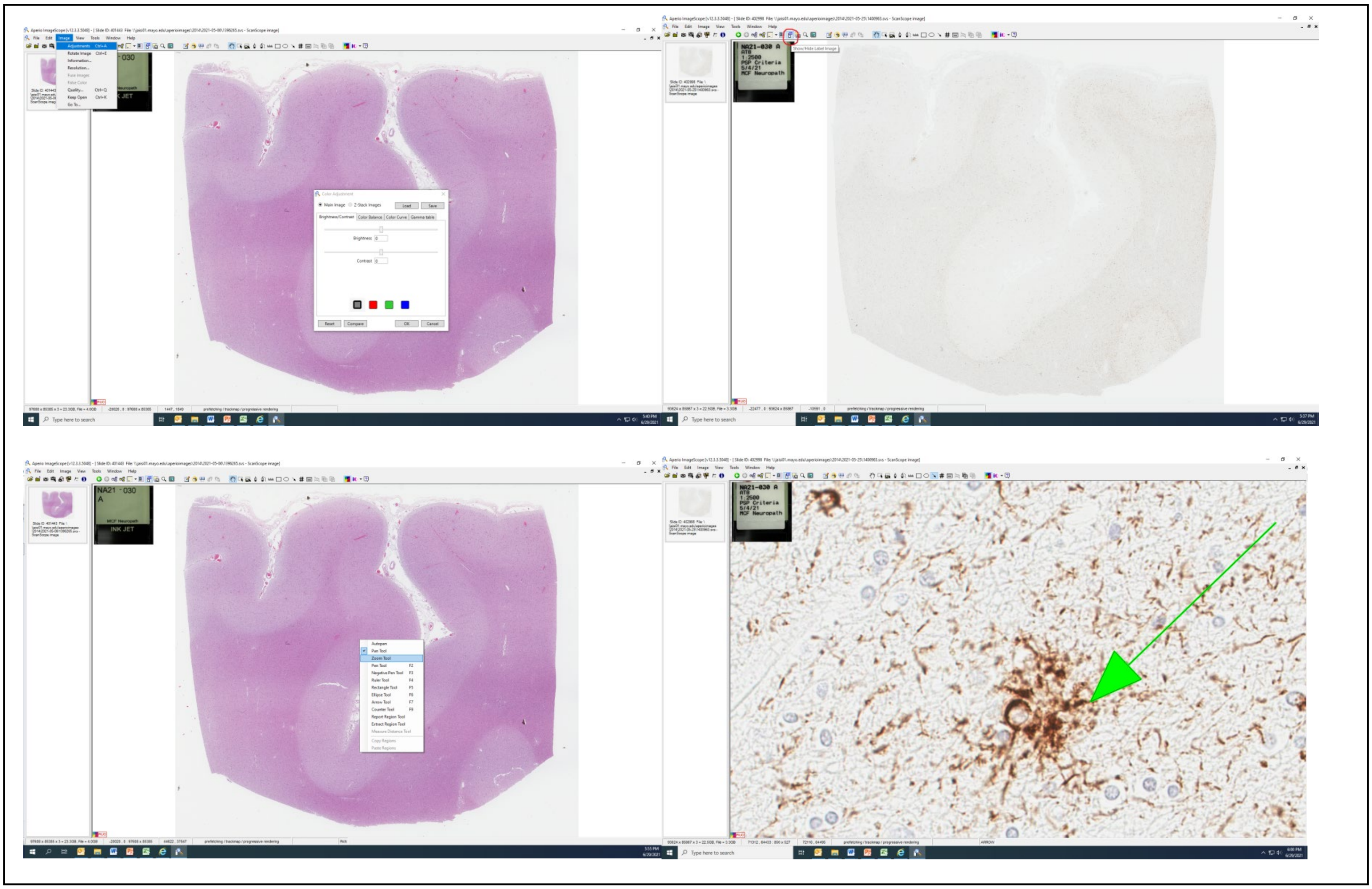

How to use the booklet

This booklet is composed of a brief description of each tauopathy. The photo atlas often covers the regions included in aim 2, and can be used as a stand-alone.

Since each slide image contains additional regions not included in the PSP criteria, these regions may contribute with unnecessary noise. Thus, when relevant, additional regions represented on the slides have also been captured in the micrographs.

Representative images have been created using phospho-tau 202 (CP13) for most images rather than AT8. The rationale, for this is the fact that all neuropathologic diagnoses were established using CP13. For study execution, AT8 was selected as a commercially available tau antibody. We acknowledge there are mild variations in the staining pattern. In some cases, appropriate work up with 3R, 4R, Gallyas silver stain and TDP-43 have been performed to confirm a diagnosis, but results have been omitted since the diagnosis for this exercise should be based on only H&E and phospho-tau stains.

Reviewing the descriptive summaries or the photo atlas are not necessarily required for completion of the study task, but are meant to provide supportive material for consultation, as needed. At any time, you may choose to skip directly to page 8 and 43-46 for the provisional neuropathologic criteria, block key, table of lesions required for the PSP diagnosis, scoring sheet and a summary of the provisional criteria.
An image of the scoring sheet is available on page 45. The associated excel sheet may be used for entries.

The descriptive summaries are case specific and not meant to fulfill standards for a compendium on tau pathology. When in doubt, you may choose to go directly to the description of the tauopathy in doubt, if any.

We thank you for participating in this study and hope you will enjoy this experience.

Sincerely,

Dennis W. Dickson, MD, Mayo Clinic, Jacksonville, FL
Shanu F. Roemer, MD, Mayo Clinic, Jacksonville, FL
Lea Grinberg, MD, PhD, University of Californian, San Francisco, CA
William W. Seeley, MD, PhD, University of Californian, San Francisco, CA
John F. Crary, MD., PhD, Icahn School of Medicine at Mount Sinai, NY
Ann C. McKee, MD, Boston University, Boston, MA

1. Anatomical regions and sampling scheme

.1 Macroscopic examination

The fixed brain weight is often within normal limits. Many cases, however, show mild atrophy of the frontal convexities (Figure 1). The atrophy may involve the precentral gyrus. The medial temporal lobe is often preserved. Atrophy of the midbrain is often evident on the external surface of the brainstem. Coronal sections through the hippocampal formation show no significant atrophy. The parietal and occipital cortices are usually grossly unremarkable. Coronal sections through the basal ganglia may show atrophy and red-brown discoloration of the globus pallidus. The subthalamic nucleus shows atrophy, often severe, sometimes with rusty red-brown discoloration. The thalamus is often macroscopically unremarkable. The midbrain shows atrophy and often dilation of the aqueduct of Sylvius. Atrophy of the superior colliculus can be striking. Sections of the midbrain at the level of the superior colliculi will reveal pallor of the decussation of the superior cerebellar peduncle, making the red nucleus more apparent. The substantia nigra shows decreased neuromelanin pigment and often rusty red-brown discoloration. The cerebellar sections often show discoloration of the dentate nucleus and often marked atrophy of the hilus of the dentate nucleus. The cerebellar folia and cerebella white matter show relative preservation.

1.2 Selective vulnerability in PSP cardinal brain regions

The presence of pathology in three cardinal brain regions (*substantia nigra*, *subthalamic nucleus* and *globus pallidus*) makes it possible to reach a diagnosis of PSP. These regions are affected by neuronal and glial tau lesions, often associated with moderate to marked neuronal loss and reactive gliosis. Cardinal nuclei are affected in both typical PSP and its variants, with marked pathology in pallido-nigro-Luysial variant. The striatum, cerebellar dentate nucleus and its efferent fibers (*i.e.,* superior cerebellar peduncle), the motor nuclei of the thalamus, as well as frontal/premotor and motor cortices are usually affected in addition to the cardinal nuclei, but show varying severity of tau pathology.

1.3 Tau pathology in PSP

The hallmark tau pathology in PSP includes both neuronal (*i. e.* pretangles and tangles) and glial (*i. e.* oligodendroglial coiled bodies and tufted astrocytes) pathology, as well as threads, which are variably derived from neuronal and glial cell processes depending upon the brain region. In previous neuropathologic criteria for PSP, tufted astrocytes were not a prerequisite, but characteristic astroglial (and to a lesser extent oligodendroglial) pathology is increasingly recognized as being critical for differential diagnosis of neurodegenerative tauopathies. Tufted astrocytes the hallmark of PSP are characteristic of PSP, and are only rarely present in other sporadic tauopathies. Tufted astrocytes are present in 97% of cases of PSP in the frontal or motor cortices, and in 99% in the striatum. One challenge is that tufted astrocytes may be less conspicuous in atypical cases of PSP due to mild pathology or atypical variants. In these cases, cardinal nuclei are similarly affected with tau lesions as in typical PSP, but tau positive astrocytes may show atypical morphology. Subcortical nuclei less often have tufted astrocytes, but show neurofibrillary tangles and pretangles with varying degree of threads and coiled bodies.

1.4. Proposed neuropathologic criteria of PSP
The following represents the minimum set of histological features to meet these proposed criteria to make a diagnosis of PSP:

1. Pretangles and neurofibrillary tangles (NFT) in 2 of 3 of the following brain regions:
   - substantia nigra (SN)
   - subthalamic nucleus (STN)
   - globus pallidus (GP)
2. Tufted astrocytes (TA) in the motor cortex (Ctx) or corpus striatum (CS).

In addition, the following features support the diagnosis of PSP

- Threads and coiled bodies in the cardinal regions
- Threads, coiled bodies and tufted astrocytes are often present in the midbrain tectum
- Pretangles and threads in the olivopontocerebellar system

In some circumstance sampling of all three cardinal brain regions may not be possible (e.g., when the subthalamic nucleus cannot be adequately assessed), two of the three regions is sufficient to meet these criteria. [Rarely occurring, mutations in the *MAPT* gene do not exclude the diagnosis of PSP when the above criteria are fulfilled.]

2. NEUROPATHOLOGY OF TYPICAL PSP

2.1. Histologic findings in typical PSP

The neocortex (middle frontal, medial temporal, precentral and post-central gyri, and medial occipital gyri) are usually histologically unremarkable on H&E. In the absence of significant co-morbid Alzheimer's type pathology there is no significant superficial spongiosis, and the neuronal populations appear normal. Microvacuolation, even in PSP cases with severe cortical pathology, is unusual. Cortical reactive astrogliosis is minimal. Presence of ballooned neurons, severe spongiosis or microvacuolation should lead to consideration of another primary tauopathy (*e.g.*, Pick's disease or CBD).

The hippocampal formation and the amygdala are often unremarkable, unless there is comorbid pathology (*e.g.*, Alzheimer type pathology or hippocampal sclerosis). The basal nucleus may reveal globose tangles and mild neuronal loss.

The globus pallidus usually has mild gliosis. Cases with severe neuronal loss and gliosis should lead to consideration of an atypical form of PSP (*i.e.,* PNLA) or another disorder. Isolated coarsely granular iron-type pigment and granular-foamy axonal spheroids can be observed in typical PSP, which are more marked in PNLA. The putamen is often unremarkable, but may have moderate gliosis.

he subthalamic nucleus often has moderate to marked neuronal loss and gliosis. Neurofibrillary tangles can often be seen. The presence of iron-type pigment and granular foamy axonal spheroids in the subthalamic nucleus should raise consideration of an atypical variant of PSP (e.g., PNLA).

The midbrain often has decreased pigment in the substantia nigra. The pars compacta shows neuronal loss with extracellular neuromelanin. Both medial and ventrolateral groups may be affected. Some remaining neurons may show mild dispersion of intraneuronal neuromelanin, a subtle sign of an early neuronal tau lesion. Other neurons show intraneuronal globose tangles. Gliosis is often moderate to severe and correlates with the degree of neuronal loss. A mild degree of extracellular iron pigment may be encountered. Myelin pallor of the medial part of the cerebral peduncle is not unusual, but selective pallor of the corticospinal tract should lead to consideration of atypical PSP or another tauopathy. Gliosis and neuronal loss is common in the red nucleus but better appreciated in the periaqueductal gray matter and superior colliculus of the tectum on H&E stains. The midline nuclei may show mild gliosis but otherwise appear unaffected on H&E stains. The aqueduct may show visible dilation even on hemi-sections of the midbrain.

The cerebellar cortex may show patchy Purkinje cell loss. The white matter rarely shows any significant pallor. If the section contains the superior cerebellar peduncle, this fiber tract will often show severe myelin pallor. The dentate may show some degree of grumose degeneration.

2.2. Phospho-tau immunohistochemistry in typical PSP

<u>*Neocortex*</u>

Immunohistochemistry with phosphorylated tau antibodies (p-tau) shows mild-moderate tangles/pretangles and many tufted astrocytes in middle frontal cortex. Coiled bodies are less frequent in both the cortex and underlying white matter. Thread pathology in the absence of Alzheimer's type pathology is usually mild.

The medial temporal cortices show less abundant tau lesions with respect to tangles and threads. This can make tufted astrocytes in the entorhinal and associated temporal cortices easier to identify. Coiled bodies are rare in both the cortex and underlying white matter.

The primary motor cortex shows the similar features, but the overall p-tau burden is more pronounced than in the middle frontal cortices. Tufted astrocytes often stand out due to the lack of concomitant secondary tau pathology.

P-tau lesions in the occipital lobe are minimal. The primary visual cortex is relatively unaffected and may show sparse pretangles but only rarely any glial lesions. The visual association cortex may show glial tau such as coiled bodies or unclassifiable astrocytic lesion, and rarely tufted astrocytes. The underlying white matter is relatively unaffected but may show minimal thread pathology and scarce coiled bodies.*Basal forebrain including amygdala*

The corticomedial amygdala may show pretangles/tangles. The basolateral amygdala may contain multiple tau lesions many resembling classic tufted astrocytes, but unclassifiable astrocytic tau lesions may also be present (e.g., Figure 9 in Atlas). The nucleus basalis of Meynert will often show minimal-mild neuronal loss and many globose tangles as well as coiled bodies. The white matter tracts at the basal forebrain level will reveal an unaffected optic tract. The anterior commissure is relatively unaffected. The internal capsule may show many threads and white matter coiled bodies.

Hippocampus at the level of the lateral geniculate nucleus

The hippocampus proper often shows normal neuronal population with mild-moderate pretangles and tangle that parallel those in primary age-related tauopathy. Occasional globose tangles and astrocytic lesions may also be present. Fornix, fimbria and temporal white matter may show mild thread pathology and mild-moderate coiled bodies.

Basal ganglia with the lentiform nucleus

Putamen shows numerous astrocytic tau lesions (tufted astrocytes). Pencil fibers variably show white matter threads, this however, can be quite pronounced. Coiled bodies and globose tangles/pretangles are variably seen in the gray matter. The globus pallidus may reveal mild neuronal loss, gliosis, cribriform changes and ARTAG of unknown significance. There are often many coiled bodies, mild-moderate neuropil threads and neurofibrillary tangles. Globus pallidus often shows a gradient decrease in tufted astrocytes compared to putamen.

Hypothalamus may show many tangles, but rarely shows any astrocytic lesions and only mild thread or coiled bodies. Although, the hypothalamus is affected not only in PSP but many other tauopathies, we will not address the involvement further.

Thalamus and subthalamic nucleus

The ventral thalamus shows relatively preserved neuronal population, but tau immunohistochemistry may reveal many tangles, threads, tufted astrocytes and coiled bodies. The thalamic fasciculus shows moderate threads and multiple coiled bodies.

The subthalamic nucleus on the other hand, shows moderate-severe neuronal loss in typical PSP cases. Globose tangles are readily evident in surviving neurons. There is marked thread pathology and some coiled bodies. In contrast to caudate and putamen tufted astrocytes are usually less conspicuous.

Midbrain (level of the superior colliculus)

The midbrain will display striking p-tau deposition in the substantia nigra and dorsal midbrain, often giving an impression of an unaffected cerebral peduncle. Higher magnification will often reveal threads and coiled bodies in the medial half of the cerebral peduncle. The corticospinal tracts may show coiled bodies and thread pathology, but severe affection with these tau positive lesions should lead to consideration of an atypical variant.

The red nucleus will show many neurofibrillary tangles, coiled bodies and threads. The substantia nigra will show many neurofibrillary tangles, in particular, globose tangles, threads and coiled bodies. Tufted astrocytes are uncommon. The midline nuclei show moderate to marked thread pathology and many tangles.

The midbrain tectum often displays all four types of tau lesions (i.e., neurofibrillary tangles, coiled bodies, tufted astrocytes and threads). In some instances, marked thread pathology may camouflage tufted astrocytes at higher power, and lower magnifications will sometimes be more helpful to reveal the tufted pattern in the setting of marked neuropil threads. The periaqueductal gray matter shows many threads and tangles.

Cerebellar cortex with dentate nucleus

The cerebellar white matter shows numerous coiled bodies. Surviving neurons show multiple tangles and threads. Some coiled bodies may be evident but tufted astrocytes are rarely identified. Tau pathology in Purkinje cells is not a feature of PSP.

Region	Tangles & pretangles	Astrocytes (tufted)	Oligodendroglia (coiled bodies)	Threads
**Superior frontal cortex**	2	2	2	1
**Motor cortex**	2	3	2	2
**Putamen**	2	3	2	1
**Globus pallidus**	2	1	2	2
**Subthalamic nucleus**	3	2	2	3
**Tectum**	2	2	3	3
**Red nucleus**	2	2	3	3
**Substantia nigra**	2	1	1	2
**Cerebellar dentate nucleus**	2	0	1	2
Basal nucleus Meynert	3	0	1	2
Ventral thalamus	2	2	2	3
Oculomotor complex	2	0	0	2
Temporal cortex	1	1	0	0
Occipital cortex	0	1	0	0
Thalamic fasciculus			3	3
Cerebellar white matter			2	2

Table 2. Median tau scores in over 1,000 cases of PSP

Semiquantitative scores based on 0 to 3-point scale (none, mild, moderate marked) for each region and lesion type; red shading corresponds to regions with marked tau pathology, orange shading to regions with moderate tau pathology, and blue shading for less affected regions. Blank cells are white matter regions that do not have neuronal lesions or tufted astrocytes.

3. NEUROPATHOLOGY OF ATYPICAL PSP

Atypical PSP cases all fulfill hallmark features of typical PSP. The degree of neuronal loss, the distribution of tau hallmark lesions, the presence of associated pigment-spheroid degeneration or globular inclusions may be less prominent or accentuated compared to typical PSP.

3.1. Cortical variant PSP

For didactic purposes we have selected PSP cortical variants with none or low Braak stage and Thal phase to showcase cortical PSP lesions. In many cases, comorbid Alzheimer's type pathology is not unusual, enhancing the impression of a cortical predominant tauopathy. We have selected cases with range in severity.

Findings on H&E

The motor cortex shows neuronal loss and superficial spongiosis or microvacuolation. The middle frontal gyrus may also be affected but to a lesser degree than motor cortex. The subjacent white matter may show myelin pallor and gliosis. The hippocampus shows normal neuronal population throughout all sectors of Ammon's horn. Grains may be present in 20% of PSP cases. The basal nucleus of Meynert has near normal neuronal population. The globus pallidus has mild gliosis. The thalamus has moderate gliosis in the ventrolateral region. The subthalamic nucleus is atrophic. The substantia nigra has marked neuronal loss associated with extraneuronal neuromelanin and gliosis. The cerebral peduncle has mild myelin pallor of the corticospinal tract. The red nucleus has severe gliosis. The decussation of the superior cerebellar peduncle shows foamy macrophages. Tectum has severe gliosis.
The cerebellum has no significant Purkinje cell loss or Bergmann gliosis. The dentate nucleus has minimal neuronal loss and gliosis. There are no overt grumose changes.

Findings on phospho-tau immunohistochemistry

The frontal and motor cortices show many tangles, coiled bodies, tufted astrocytes and threads. Tufted astrocytes and thread pathology is more pronounced in the motor cortex. The temporal cortex shows sparse tangles, coiled bodies and tufted astrocytes. There is no significant thread pathology. The occipital lobe show mild tau pathology with pretangles and coiled bodies but no tufted astrocytes.

The corticomedial amygdala shows sparse pretangles and threads. The basolateral amygdala has sparse tufted astrocytes. In case of concomitant AGD, ballooned neurons and granular fuzzy astrocytes may also be noted. The basal nucleus has mild-marked tangle pathology and a few coiled bodies and threads. The ventral thalamus has marked pathology with many tangles, coiled bodies and threads. Tufted astrocytes are also seen but are less frequent.

The thalamus has many neuronal and glial lesions. The thalamic fasciculus and the posterior limb of the internal capsule have many threads and coiled bodies. This pathology extends into the corticospinal tract of the cerebral peduncle.

3.2. PSP with globular inclusions (*4R tau positive "Pick bodies" and PSP with corticospinal tract involvement, GGT type 2*)

Rarely, atypical PSP may show ballooned neurons and neuronal inclusions that may resemble Pick bodies on H&E. The presence of neuronal loss in PSP cardinal nuclei, however, hints the underlying diagnosis. Sometimes inclusions may be visible in atypical astrocytes. Phospho-tau reveals a range from tufted astrocytes to atypical astrocytes with globular inclusions. The distribution pattern, however, is similar to the distribution typical of PSP. Variable globular astrocytic and/or oligodendroglial inclusions may warrant a work up with special stains to rule out Pick's disease or globular glial tauopathies.

3.3. Hindbrain predominant PSP

Typical PSP and hindbrain predominant both show hallmark pathology in cardinal nuclei. In hindbrain predominant PSP neuronal loss and hallmark tau lesions are often mild in forebrain structures. On the other hand, severe PSP and hindbrain predominant PSP may both show severe grumose changes of the cerebellar dentate nucleus evident on H&E. Despite relative neuronal preservation on H&E, phospho-tau may reveal many pretangles and tangles. As is the nature of PSP, motor cortex may still show moderate-marked affection despite relative sparing of other cortical regions. Due to the severe neuronal loss, tau lesions in the cerebellar dentate may be less conspicuous and not show significant differences to typical PSP.

3.4. PSP with pallido-nigral-Luysial atrophy (PSP-PNLA)

Typical PSP and PSP-PNLA both show hallmark pathology in cardinal nuclei. Furthermore, both may show gliosis associated with variable degree of iron-type pigment and granular-foamy spheroids in the globus pallidus, subthalamic nucleus and substantia nigra. These features, however, often are more prominent in PSP-PNLA. Similarly, atrophy of pallido-nigral-Luysial regions is often more severe than in typical PSP.

3.5. Differential diagnostic consideration

The oligodendroglial lesions in atypical PSP predominantly show typical features of coiled bodies rather than large globular inclusions. Individual astrocytes may show globular feature but again the predominant finding is tufted astrocytes. It remains to be validated whether atypical PSP such as cortical variant PSP with corticospinal tract affection and globular glial tauopathy type 2 are a spectrum of the same disease or distinct entities as previously proposed. Although a clinical diagnosis of CBD is not unusual in cortical variant PSP, the presence of ballooned neurons should warrant a consideration of concomitant AGD, especially if these are mainly found in limbic regions and astrocytic plaques are inconspicuous. Diagnostic work up also excluded a primary motor neuron disease. Similarly, it remains to be validated whether PSP with globular neuronal inclusions (Pick body like) and GGT are truly distinct disorders or part of a disease spectrum. GGT type II and atypical PSP may overlap significantly, raising the need for more sensitive terms of differentiating the tau pathologies in addition to routine neuropathologic work-up. In most instances, genetic testing will not reveal any known pathogenic mutation in *MAPT* although this may also be a differential diagnostic consideration.

4. OTHER TAUOPATHIES

4.1. Globular glial tauopathy Type 1

Findings on H&E

The frontal and medial temporal cortices are often remarkable for neuronal loss and gliosis. In areas with the most severe cortical pathology, white matter changes are evident with myelin pallor and gliosis. The precentral and post-central cortices and occipital cortices are relatively preserved. The hippocampal formation is affected, particularly the subiculum may show marked neuronal loss and gliosis. The amygdala has neuronal loss and gliosis. The basal nucleus of Meynert has minimal neuronal loss. Amphophilic-basophilic globular glial inclusions may be identified in the white matter, and are often readily visible in the white matter bundles of the basal forebrain (anterior commissure, internal capsule, pencil fibers). The globus pallidus and putamen are relatively unremarkable. So is the subthalamic nucleus. On the other the substantia nigra has moderate neuronal loss and gliosis, including the ventrolateral cell groups. The red nucleus has mild gliosis. The cerebral peduncle shows mild pallor of the frontobulbar tract. The cerebellar dentate nucleus is well preserved and free of grumose changes.

Findings with phospho-tau immunohistochemistry

The frontal and medial temporal cortices are particular affected and show numerous globular glial inclusions. There are pretangles and tangles but more prominent thread pathology. The underlying white matter shows numerous globular oligodendroglial inclusions and similarly has prominent thread pathology. The precentral and postcentral cortices are less affected but show similar tau lesions. The occipital cortices are relatively spared.

The amygdala shows many globular glial inclusions (oligodendroglial and astrocytic). The basal nucleus of Meynert has pretangles and many threads and globular oligodendroglial inclusions. The anterior commissure and internal capsule has many globular glial inclusions and marked thread pathology. The putamen and globus pallidus show similar inclusions.

The subthalamic nucleus is relatively spared and only shows minimal glial inclusions. The lenticular striate fibers and the thalamic fasciculus, however, show many globular inclusions and moderate thread pathology.

The substantia nigra has pretangles and tangles, threads and globular glial inclusions. The red nucleus and midbrain tectum show milder pathology. The cerebral peduncle is affected with the frontobulbar tract more affected than the corticospinal tract.

The cerebellar white matter and dentate nucleus are mostly spared and only show isolated tau lesions.

Overall, although, various types of glial inclusions are encountered, globular oligodendroglial inclusions are far more conspicuous than globular astrocytic inclusions and coiled bodies.

4.2. Globular glial tauopathy Type 2

The current evidence suggests that GGT Type 2 is a variant of PSP with marked involvement of frontal and motor cortices with corticospinal tract degeneration. See **Section 3.2** on atypical PSP with globular glial inclusions.

4.3. Globular glial tauopathy Type 3

Findings on H&E

The frontal and motor cortices have mild neuronal loss and gliosis. The medial temporal, parietal and occipital cortices are relatively unremarkable. So is the underlying white matter, but incidental leukoaraiosis. The hippocampus has gliosis, neuronal loss and mild microvacuolation, particularly in the subiculum. The amygdala has moderate gliosis and neuronal loss. The basal nucleus of Meynert has minimal neuronal loss. The globus pallidus and the putamen are relatively unremarkable except for cribriform changes. Globular glial inclusions are best appreciated in the anterior commissure. The subthalamic nucleus is unremarkable, but shows many artifactual perineuronal vacuoles. On the other hand, the substantia nigra has moderate neuronal loss, most marked in the ventrolateral cell groups. The red nucleus and midbrain tectum have mild gliosis. The cerebral peduncle is unremarkable. The cerebellum show no myelin patter or grumose changes.

Findings on phospho-tau immunohistochemistry

The frontal and the motor cortices have prominent globular astrocytic lesions with the latter showing a striking involvement ('carpet-like') compared to the opposing postcentral gyrus. The medial temporal cortices also show marked tau lesions with many globular astrocytic lesions. The occipital cortices show involvement with mild glial lesions.

The hippocampus has numerous pretangles in addition to astrocytic lesions. The amygdala has marked tangles, threads and globular glial inclusions. The basal nucleus of Meynert show many globular glial inclusions and threads but less conspicuous tangle pathology. The putamen has numerous globular astrocytic lesions. The globus pallidus is less affected but also has globular glial inclusions and pretangles.

The thalamus and subthalamic nucleus have many pretangles, tangles and globular glial inclusions. Thread pathology is mild-moderate. The red nucleus and midbrain tectum are also affected.

The substantia nigra has tangles and threads as well as many globular glial inclusions. The cerebral peduncle shows involvement of both frontobulbar and corticospinal tract although globular inclusions are more frequent in the latter.

The cerebellum shows mild pathology with pretangles and tangles in the dentate nucleus and only mild glial lesions and threads in the white matter.

The marked involvement of the amygdala is unexpected in GGT type 3, but nevertheless, throughout, globular astrocytic inclusions are far more conspicuous than globular oligodendroglial inclusions and coiled bodies and the severe involvement of the frontotemporal, the motor cortex and corticospinal tract would is consistent with GGT type 3.

4.4. Corticobasal degeneration

Pathologically both PSP and CBD are associated with neuronal and glial filamentous lesions. The distribution and differences in tau lesions including astrocytic tau, is used to differentiate CBD from PSP. Tau positive astrocytic plaques are characterized by clusters of short, irregular tau immunoreactivity in the cell processes surrounding the unstained cell body giving the pseudo-impression of a small central clearing in some astrocytic plaques. The nucleus of individual astrocytes may be difficult to identify in cases with severe superimposed thread pathology. The white matter often shows marked thread pathology affecting U-fibers and subjacent white matter. There are often many coiled bodies.

Findings on H&E

The neocortex often shows marked superficial spongiosis, although not always the case. When present, this is often associated with conspicuous neuronal loss, microvacuolation and gliosis especially in cortical layer II, but microvacuolation may also be pronounced in lower cortical layers. Ballooned neurons are encountered in most cases, but may be sparse in frontal, precentral and postcentral cortices and in cases with severe neuronal loss. Cases with severe atrophy may have sampling of association cortices that will show similar changes. The subjacent white matter has rarefaction, gliosis and loss of myelinated fibers evident on H&E sections. In the absence of significant Alzheimer's type pathology, the hippocampus usually has preservation of neuronal populations in all sectors of Ammon's horn. The amygdala and basal nucleus of Meynert are unremarkable. The globus pallidus may show mild neuronal loss and gliosis. The putamen is usually unremarkable. The thalamus and subthalamic nucleus may have mild gliosis. The substantia nigra will often reveal gliosis and moderate to severe and neuronal loss with extraneuronal neuromelanin. The neuronal loss is often most severe in ventrolateral cell groups, but dorsal and medial cell groups may also be affected. The red nucleus and tectum may have gliosis. The cerebral peduncle is unremarkable.

Findings on phospho-tau immunohistochemistry

The neocortex often shows many tangles and pleomorphic inclusions in small neurons in the cortical layer II. Densely stained globose tangles are rare, although ballooned neurons may reveal dense tau staining. There is marked thread pathology. In areas where these are most pronounced, astrocytic plaques may be harder to tease out. Cortical oligodendroglial coiled bodies may be present but these are sparse compared to PSP. All cortical layers are affected by moderate to marked tau positive threads. The underlying white matter may be severely affected with tau threads and various degree of coiled bodies. The occipital cortices may be relatively spared or show pretangles and mild threads and astrocytic tau pathology. The underlying white matter is relatively spared of tau threads. Medial and inferior temporal cortices may show threads and astrocytic plaques.

The hippocampus may reveal many astrocytic plaques in the pyramidal cell layer. The amygdala may have many pretangles and tangles and astrocytic plaques, the latter are especially predominant in the basolateral amygdala. The basal nucleus of Meynert has many pretangles and tangles and threads and does not offer much information with respect to the differential diagnosis of PSP. The anterior commissure may have mild thread pathology but only isolated glial lesions.

Globus pallidus has pretangles, tangles and threads, but coiled bodies are often less frequently encountered than in PSP. Putamen often has more marked thread pathology than seen in PSP.

The substantia nigra is remarkable for many neuropil threads, some coiled bodies but often does not show any astrocytic plaques. The residual nigral neurons may show globose neurofibrillary tangles or corticobasal bodies. The red nucleus has mild-moderate threads and tangles. The midline nuclei have threads and tangles. The midbrain tectum has many tangles, and glial tau lesions with both coiled bodies and astrocytic plaques. The cerebral peduncle may reveal many tau positive threads and coiled bodies. The temporoparietal tracts are often more affected than the frontobulbar tracts.

The cerebellar white matter is often unaffected, and may aid in the differential diagnosis of PSP. The dentate nucleus, however, may show mild pretangles, tangles and neurites.

4.5. Argyrophilic Grain disease

Findings on H&E

In the absence of significant Alzheimer's type pathology, all included study regions generally look unaffected on H&E.

Findings on phospho-tau immunohistochemistry

Tau lesions are minimal in frontal, precentral or postcentral and occipital cortices, showing isolated neurites, grains, coiled bodies, tau positive astrocyte or pretangles only. The medial temporal cortices, on the other hand may show moderate-marked grain pathology, glial lesions and threads. Coiled bodies can easily be identified in the underlying white matter.

The hippocampus shows many pretangles, tangles and grains in the subiculum. The endplate has dendritic dystrophy, a feature that may also be encountered in chronic traumatic encephalopathy. The amygdala shows many grains, accompanied by many tau positive ballooned neurons and granular fuzzy astrocytes. The corticomedial amygdala is more severely affected than the basolateral amygdala. The basal nucleus of Meynert has pretangles or tangles but more numerous coiled bodies. The anterior commissure has coiled bodies.

The globus pallidus is relatively spared, especially with respect to threads, but shows some coiled bodies. The putamen has only mild thread pathology and coiled bodies. The pencil fibers are relatively spared. Isolated astrocytic lesions may be seen. The thalamus and subthalamic nucleus may show mild tau pathology, mainly with sparse coiled bodies and granular fuzzy astrocytes. A few tangle and pre-tangles may be seen but these are not associated with any neuronal loss.

Substantia nigra is relatively unaffected but may show a few aging related tangles. Red nucleus may show isolated tangles and threads. The tectum and white matter tracts may show isolated coiled bodies and granular fuzzy astrocytes. The cerebellum is relatively spared, but may show an isolated pretangle or glial lesion.

4.6. Pick's disease

Findings on H&E

The frontal and premotor cortices show severe neuronal loss, spongiosis and gliosis. Pick bodies can often be identified in residual neurons, but ballooned neurons may be more difficult to identify due to the severe neuronal loss. The underlying white matter is severely affected showing extensive myelin fiber loss and reactive astrogliosis. U-fibers may focally be spared. The primary motor cortex is relatively preserved. The medial temporal cortices are severely affected and demonstrate ballooned neurons and Pick bodies in addition to superficial spongiosis and neuronal loss.

The hippocampus has neuronal loss in the Sommers's sector and in the dentate fascia. There are numerous Pick bodies in remaining neurons, clearly exceeding the frequency of other tauopathies with Pick body-like inclusions. The amygdala has severe neuronal loss, gliosis and microvacuolation. Pick bodies are present in many residual small neurons. A few ballooned neurons can be identified. The basal nucleus of Meynert is well-populated and show tangle pathology but not any readily visible Pick bodies.

The globus pallidus and the posterior putamen are relatively unremarkable. The thalamus has gliosis in the anterior and dorsomedial nuclei with better preservation of the lateral and ventral areas. The subthalamic nucleus is relatively unaffected, except for vascular changes.

The substantia nigra is well-populated in all cell groups and only show mild neuromelanin incontinence. The red nucleus is unremarkable. The midbrain tectum has gliosis and individual neurons with Pick bodies. The cerebellar white matter and dentate nucleus are generally unremarkable.

Findings on phospho-tau immunohistochemistry

The frontal cortices show severe tau pathology with ballooned neurons, threads and glial inclusions. Due to the severe pathology, some Pick bodies in small neurons may resemble glial cells with large globular inclusions. The underlying white matter is severely affected by threads and small globular oligodendroglial inclusions (Pick body like). Pick bodies are more easily identified in the less severely affected premotor cortex. The motor cortex, nevertheless, shows many astroglial lesions as well as moderate thread pathology. Pick bodies can easily be identified in many neurons of the pre-postcentral gyri. The white matter is affected.

The occipital cortices may have Alzheimer's type pathology.

The medial temporal cortices are severely affected by Alzheimer's type pathology. In addition there are numerous Pick bodies and astroglial lesions. The subjacent white matter is severely affected by threads and small globular oligodendroglial inclusions. Some coiled bodies can also be seen.

The hippocampus may have Alzheimer's type pathology in addition to widespread Pick bodies in many residual neurons of the pyramidal cell layer and dentate fascia. The amygdala is severely affected by Alzheimer's type pathology and Pick bodies.

Globus pallidus has marked thread pathology, pretangles and tangles. Some neurons have Pick bodies. Putamen is severely affected and show moderate-marked thread pathology. All pencil fibers are also affected.

Thalamus has marked thread pathology with pleomorphic inclusions, including Pick bodies. The subthalamic nucleus is also affected.

Substantia nigra has threads and neuronal inclusions. The red nucleus has threads, neuronal inclusions and a few tangles. The midbrain tectum has marked thread pathology and many Pick bodies. The cerebral peduncle has threads in the temporopontine and frontobulbar tracts. The corticospinal tract is less affected.

There is mild thread pathology in the cerebellar white matter. The dentate nucleus is relatively spared but has a few pretangles and threads.

4.7. Chronic traumatic encephalopathy

Findings on H&E

The neocortices, basal forebrain, subcortical nuclei, brainstem and cerebellum all show variable degrees of neurodegeneration. In some cases hemosiderin and microbleeds may be present. A subset of patients may have evidence of remote contusions.

Findings on phospho-tau immunohistochemistry

The lack of a stereotypic involvement of the affected anatomic regions combined with many NFTs and patchy perivascular deposits of NFTs and p-tau positive astrocytes will often give away the diagnosis. Although the depth of the sulci is a hallmark feature in CTE, this feature may not be present on all sections with cortex represented due to the sampling favoring anatomic regions vulnerable to PSP pathology. Tufted astrocytes and coiled bodies are absent to rare.

4.8. Tauopathies associated with *MAPT* mutations

In this study we have selected two cases with the N279K variant representing a *MAPT* mutation. One patient had a well characterized family history, the other had none.

Findings on H&E

For both cases, atypical features were present. Although, mild superficial microvacuolation, gliosis and a few ballooned neurons may be visible on H&E in the neocortex, this is rarely to the same degree as in many cases of CBD. The globus pallidus and subthalamic nucleus show neuronal loss and gliosis. Substantia nigra has neuronal loss with extraneuronal neuromelanin. The cerebellum is unremarkable.

Findings on phospho-tau immunohistochemistry

Oligodendroglial coiled body like lesions, marked thread pathology and the presence of ballooned neurons raises the differential diagnosis of CBD. Some oligodendroglia had globular inclusions. Astrocytic pathology was atypical and most frequently showed resemblance with astrocytic plaques, although some may argue that individual lesions resembled tufted astrocytes or even astrocytes with globular glial inclusions.

Based on the distribution of pathology, the two cases were signed out with a diagnosis of pallido-ponto-nigral degeneration and PSP respectively.

Photo Atlas

Progressive Supranuclear Palsy

(Typical and Atypical PSP)

And Other Tauopathies

Index

Typical PSP and atypical PSP, features on H&E (figure 1)	p. 20
Typical PSP, features on phospho-tau (figures 2-4)	p. 21-23
Atypical PSP, PSP-PNLA on phospho-tau (figures 5-6)	p. 24-25
Atypical PSP with globular inclusions (Pick like), features on H&E (figure 7)	p. 26
Atypical PSP with globular inclusions (Pick like), features on phospho-tau (figure 8)	p. 27
Atypical PSP with globular inclusions (GGT type 2), features on phospho-tau (figure 9-10)	p. 28-29
Globular Glial tauopathy type 1 and type 3 on H&E (figure 11)	p. 30
Globular Glial Tauopathy type 1 (figure 12-13)	p. 31-32
Globular Glial Tauopathy type 3 (figure 14-15)	p. 33-34
Corticobasal degeneration (figure 16-17)	p. 35-36
Argyrophilic grain disease (figure 18)	p. 37
Pick's disease (figure 19-20)	p. 38-39
Chronic Traumatic Encephalopathy (figure 21)	p. 40
Tauopathies with known MAPT mutation (figure 22-23)	p. 41-42

Typical PSP and variants

Figure 1

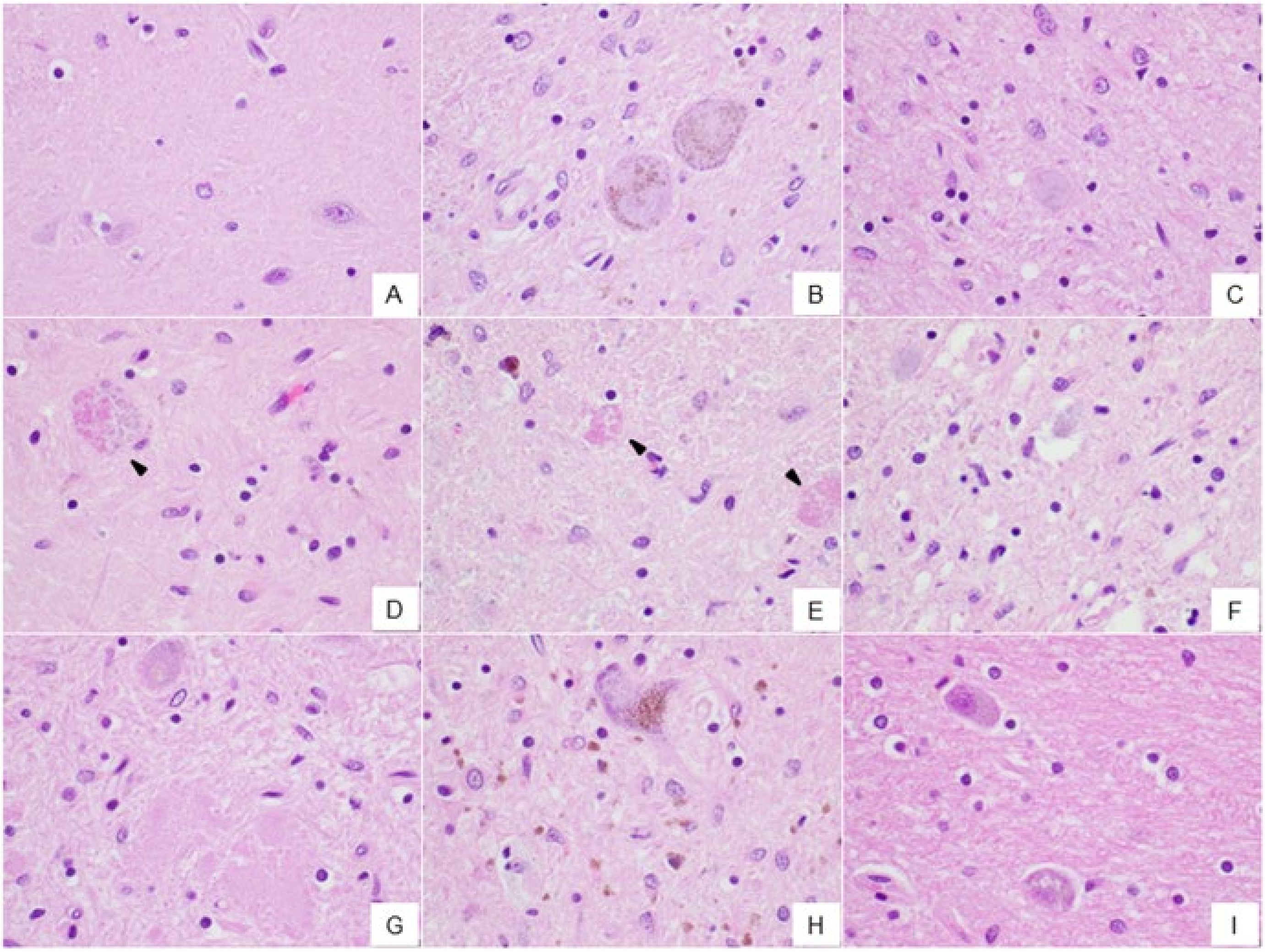

A-C. Typical PSP. A. Globus pallidus with mild gliosis and relative neuron preservation. B. Substantia nigra with globose tangles and gliosis. C. Subthalamic nucleus with globose tangle and moderate astrogliosis. **D-F.** PSP-PNLA. D. Globus pallidus with neuronal loss, gliosis and granular foamy spheroid. E. Substantia nigra with neuronal loss. Arrowheads; granular foamy spheroids. F. Subthalamic nucleus with severe neuronal loss, gliosis and myelin pallor. **G-I.** Hindbrain predominant PSP. G. Cerebellar dentate nucleus with grumose degeneration, gliosis and neuronal loss. H. Substantia nigra with severe neuronal loss. I. Subthalamic nucleus with relative myelin preservation and neuronal population.

Typical PSP

Figure 2

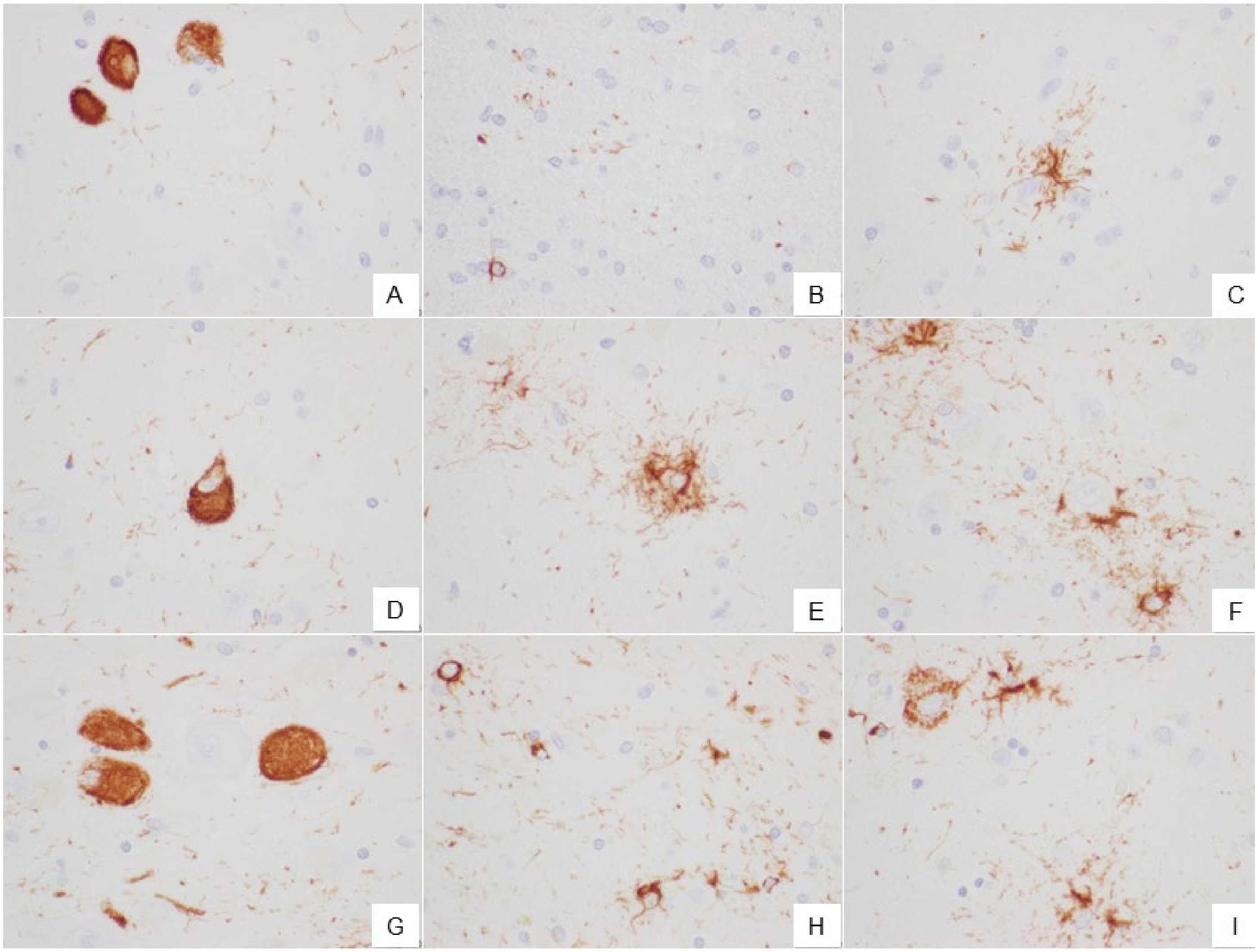

A. Globose tangles and threads in the hippocampus (subiculum). B. White matter (fornix) with coiled bodies and threads. C. Temporal association cortices (entorhinal) with tufted astrocytes. D. Amygdala with globose tangles and E. Tufted astrocytes. F. Processes from tufted astrocytes may form a syncytium. G. Basal nucleus of Meynert with many globose tangles and threads. H. Globus pallidus with many coiled bodies. I. Lentiform nucleus (putamen) with pretangles, coiled bodies and many tufted astrocytes.

Typical PSP

Figure 3

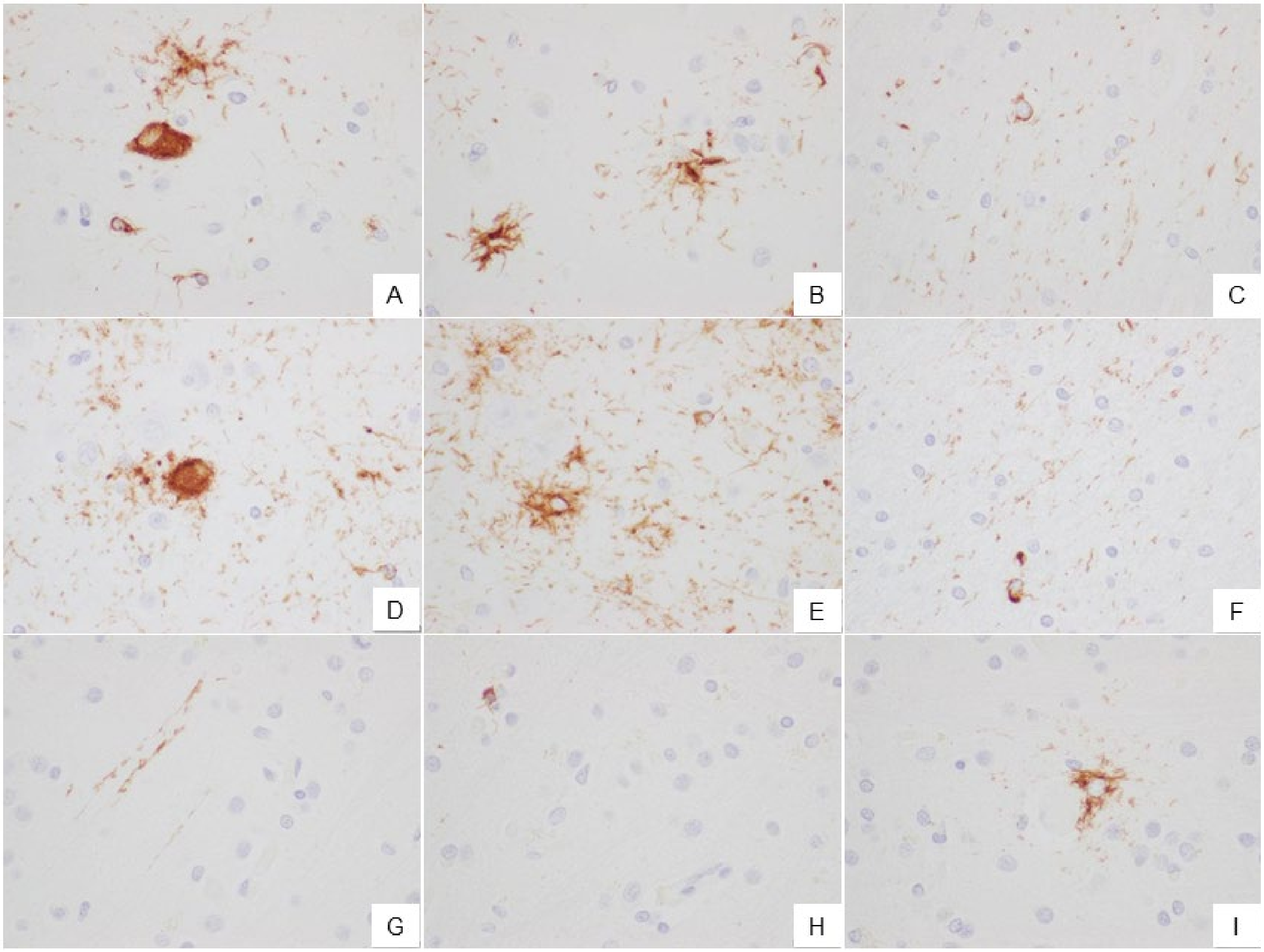

A-C; Frontal association cortices (middle frontal) often show the typical triad of tau lesions.
D-F; Primary motor cortex often show moderate-severe tau pathology.
G-I; Primary visual cortex is often relatively spared and rarely show more than one type of tau lesion within a microscopic field.

Typical PSP

Figure 4

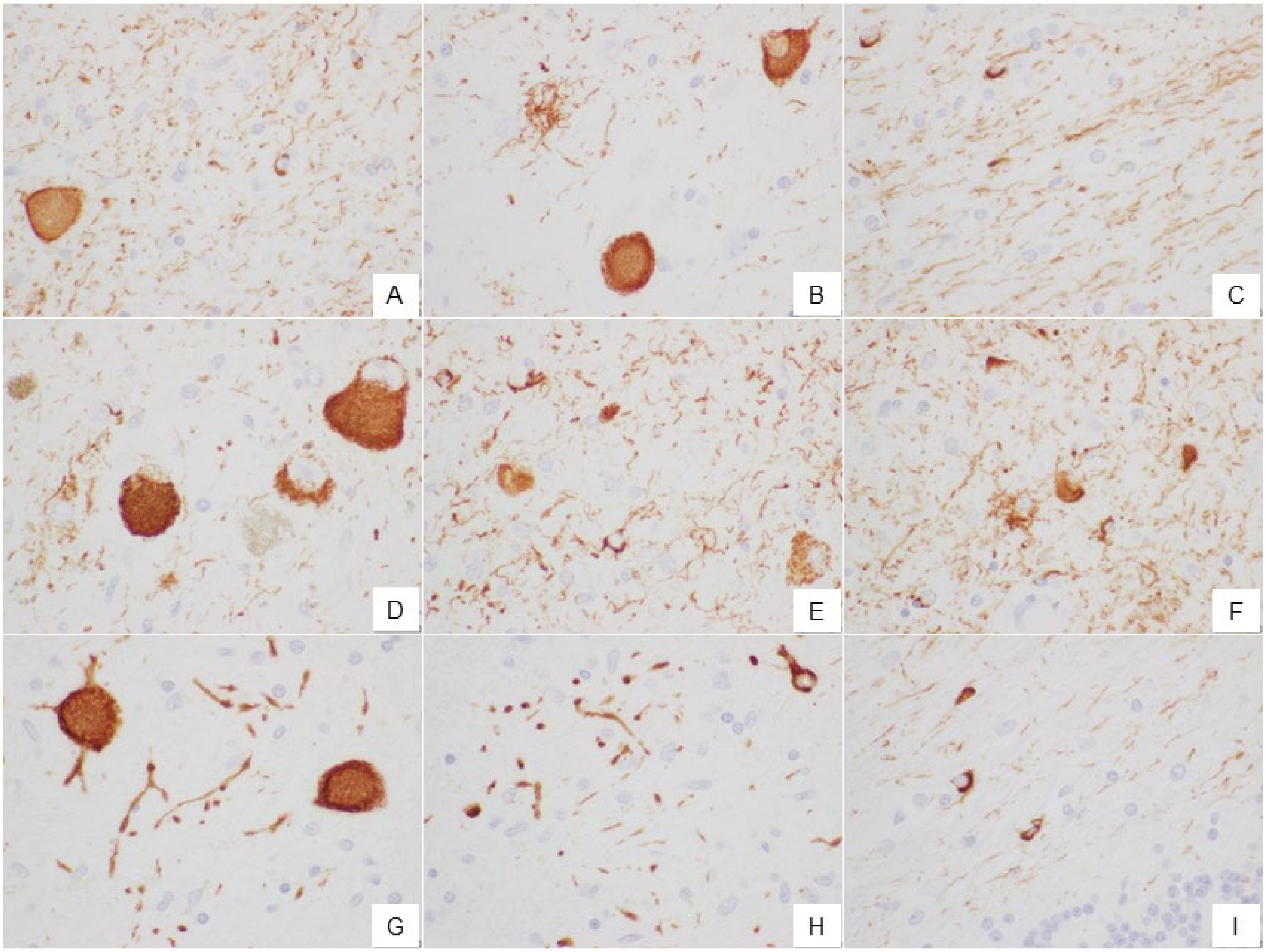

A. Subthalamic nucleus with threads, tangles and coiled bodies. B. Thalamus with pretangles and tangles, threads and tufted astrocyte. C. Thalamic fasciculus with threads and coiled bodies. D. Substantia nigra with pretangles and tangles and threads. E. Red nucleus with pretangles, coiled bodies and threads. F. Superior colliculus with threads, coild bodies, pretangle and astrocytic lesion. G. Dentate nucleus with globose tangles and threads. H. Dentate nucleuss with threads and coiled body. I. Cerebellar white matter with threads and coiled bodies.

Atypical PSP-PNLA

Figure 5

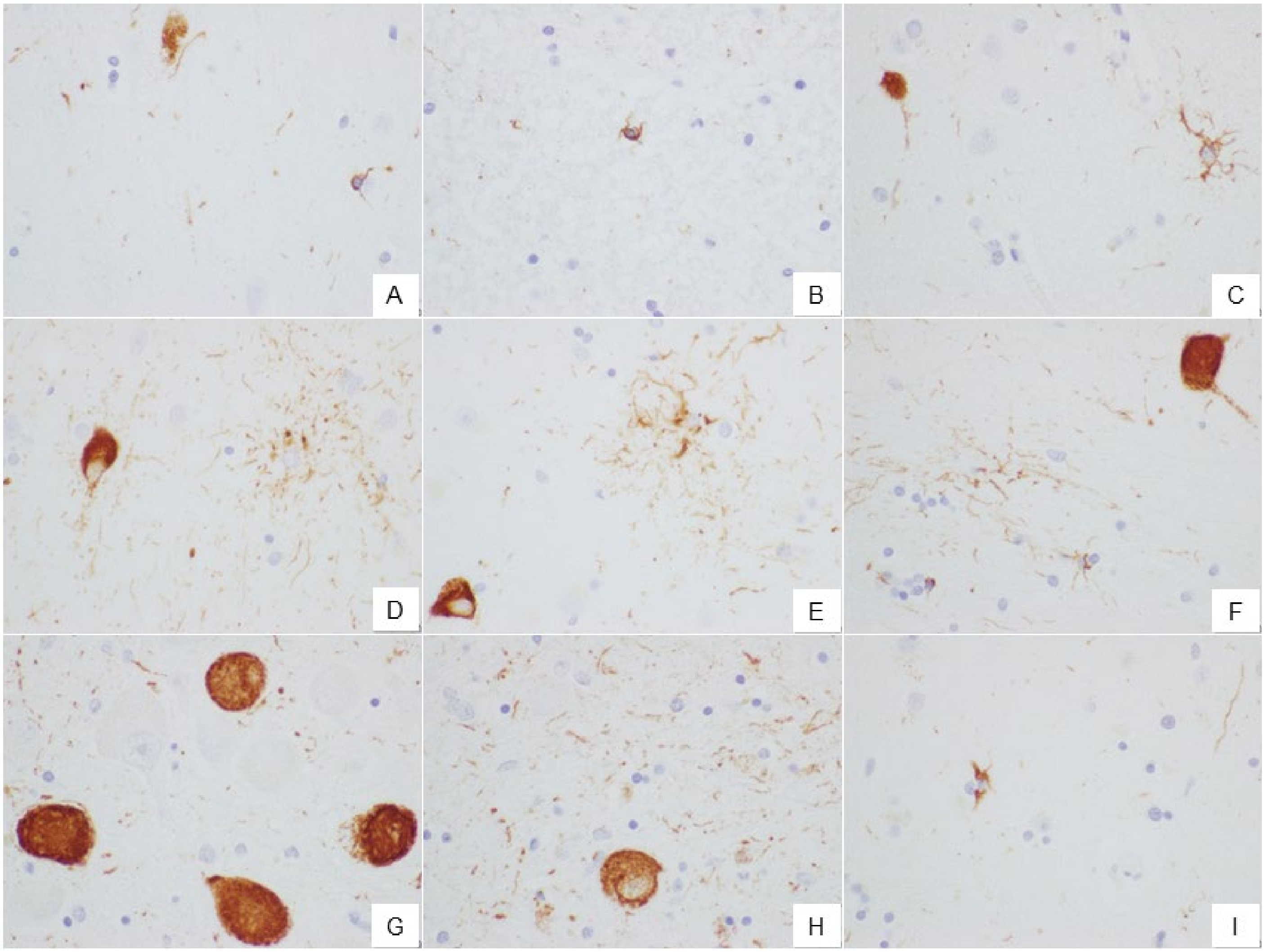

A. Subiculum with sparse pathology, pretangle and coiled body. B. Fimbria with coiled body. C. Entorhinal with pretangle and astrocytic lesion. D. Corticomedial amygdala with tangle and ill-defined astrocytic lesion. E. Basolateral amygdala with tangle and tufted astrocyte. F. Basolateral amygdala with tangles, threads and coiled bodies. G. Basal nucleus of Meynert with tangles. H. Globus pallidus with threads and a tangle in a residual neuron. I. Putamen with glial lesion

Figure 6

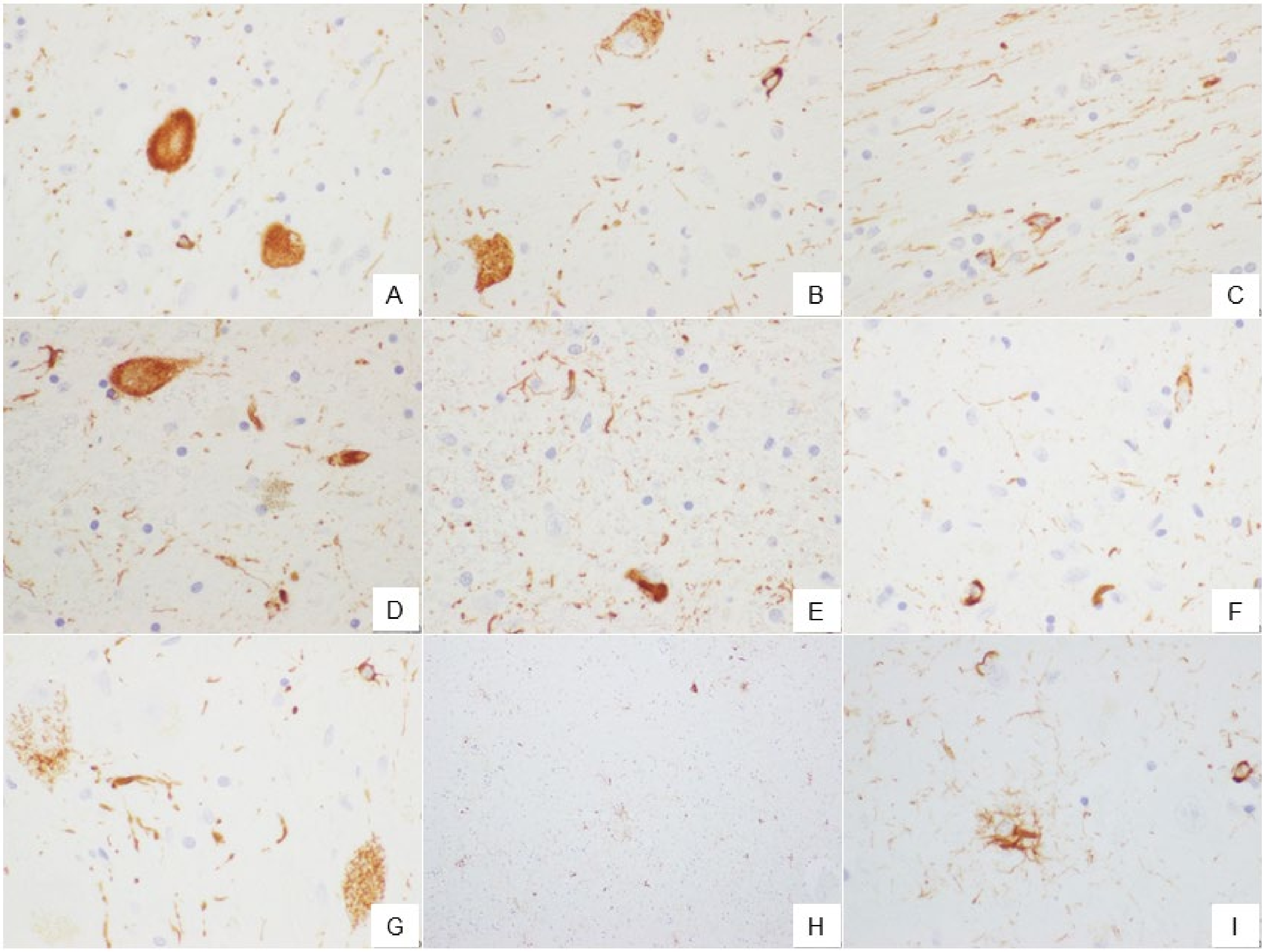

A. Subthalamic nucleus with severe with pretangles and tangles, threads and coiled body. B. Thalamus with threads, pretangles and coiled body. C. Thalamic fasciculus with threads and coiled bodies. D. Substantia nigra with pretangles and threads. E. Redu nucleus with threads and pretangle. F. Superior colliculus with mild thread pathology and coiled bodies. G. Dentate nucleus with pretangles and threads. H. Low power of the motor cortex showing mild-moderate tau pathology (x10). I. Higher power of the motor cortex showing tufted astrocyte, threads and ciled body (A-G, I x60).

Atypical PSP-With Globular Inclusions

Figure 7

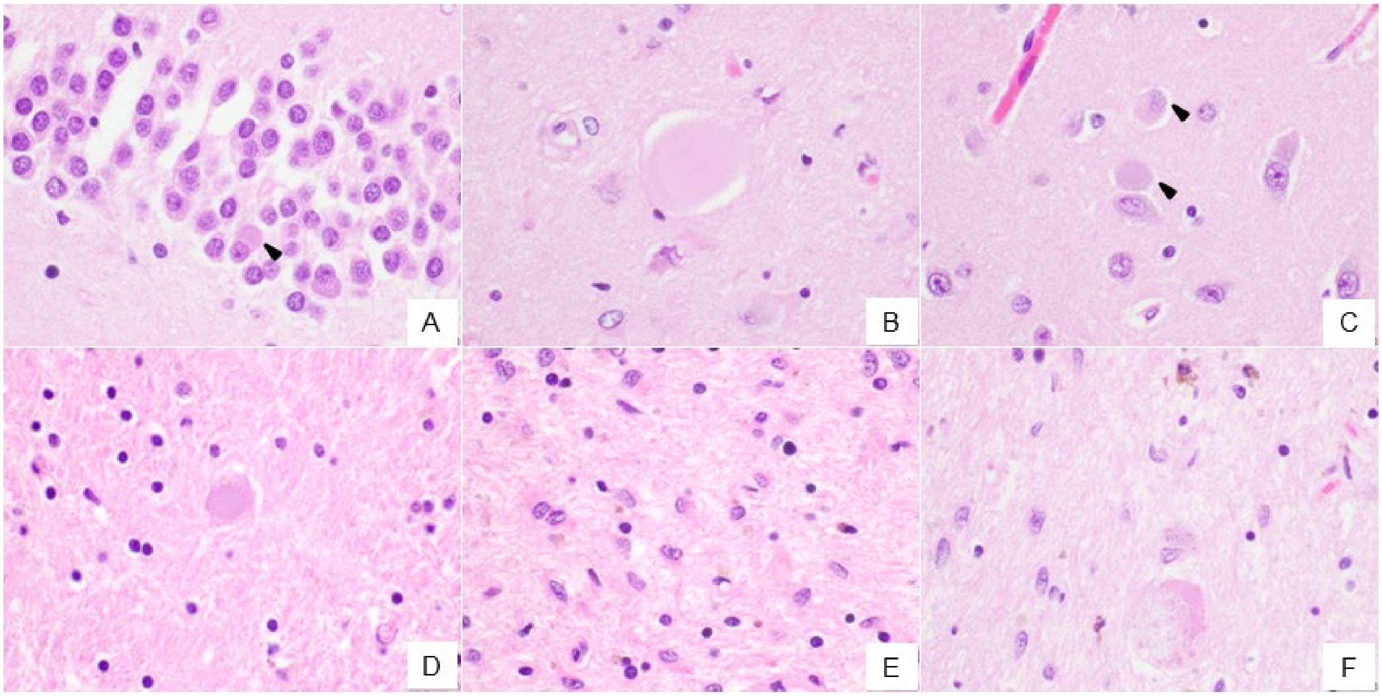

A. Dentate fascia with Pick body like inclusion (arrowhead). B. Amygdala with ballooned neuron. C. Amygdala with Pick body like inclusions (arrowheads). D. Globus pallidus with neuronal inclusion. E. Subthalamic nucleus with severe neuronal loss and gliosis. F. Substantia nigra with severe neuronal loss, gliosis, pigment and granular foamy spheroid.

Atypical PSP-With Globular Inclusions

Figure 8

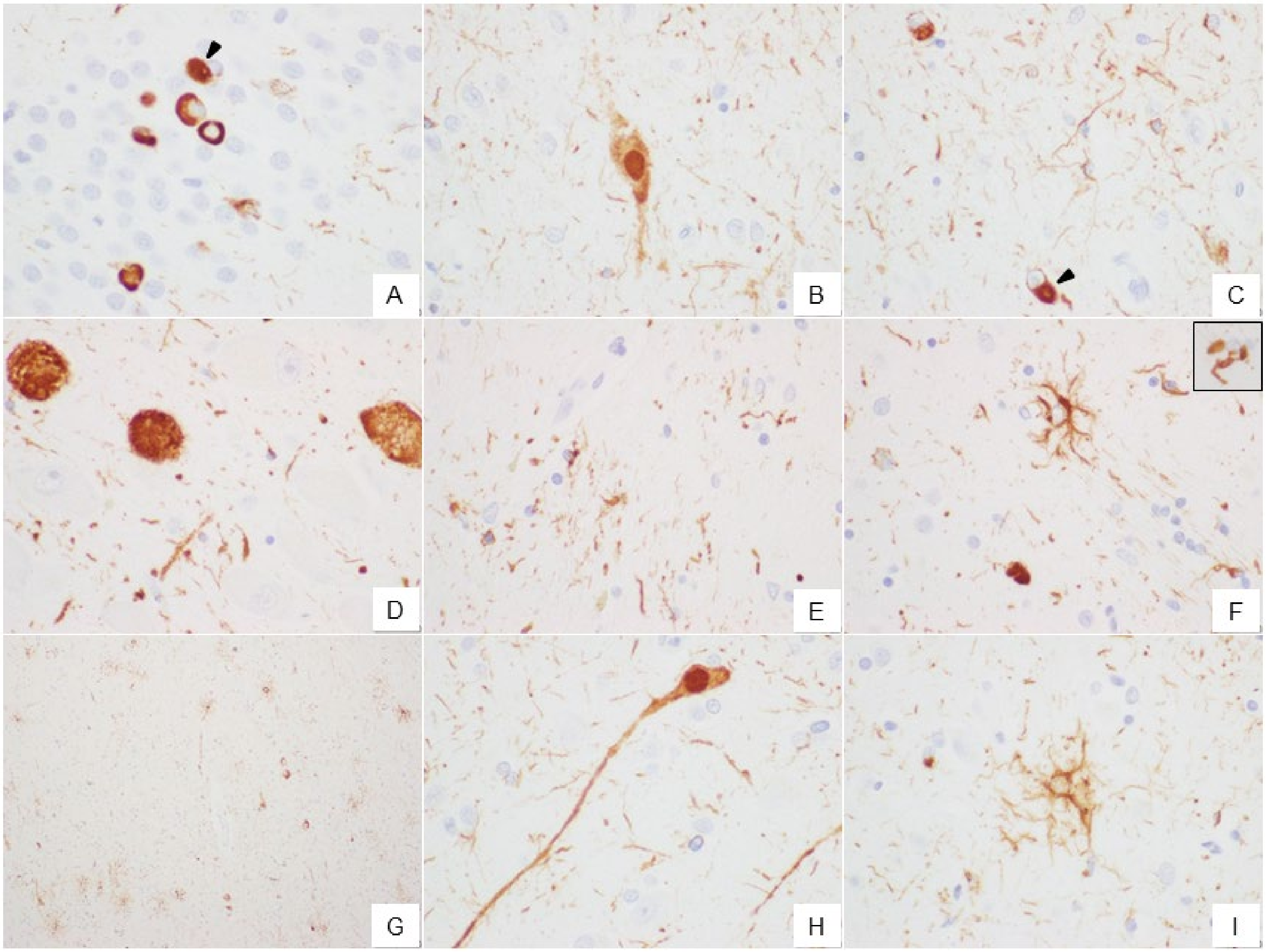

A. Dentate fascia with Pick body like inclusion (arrowhead) and tangles in granular cell neurons. B Amygdala with globular neuronal inclusion. C. Amygdala with Pick body like inclusion (arrowhead) and tangle in a small sized neuron. D. Basal nucleus of Meynert. E. Globus pallidus. F. Putamen inset with atypical globular glial inclusions. G-I. Motor cortex, G. many tau positive astrocytes and pretangles and tangles (x10). H. Occasional neurons show globular inclusions. I. Tufted astrocyte. (A-F, H-I, x60)

Atypical PSP Globular Glial Inclusions-GGT Type 2

Figure 9

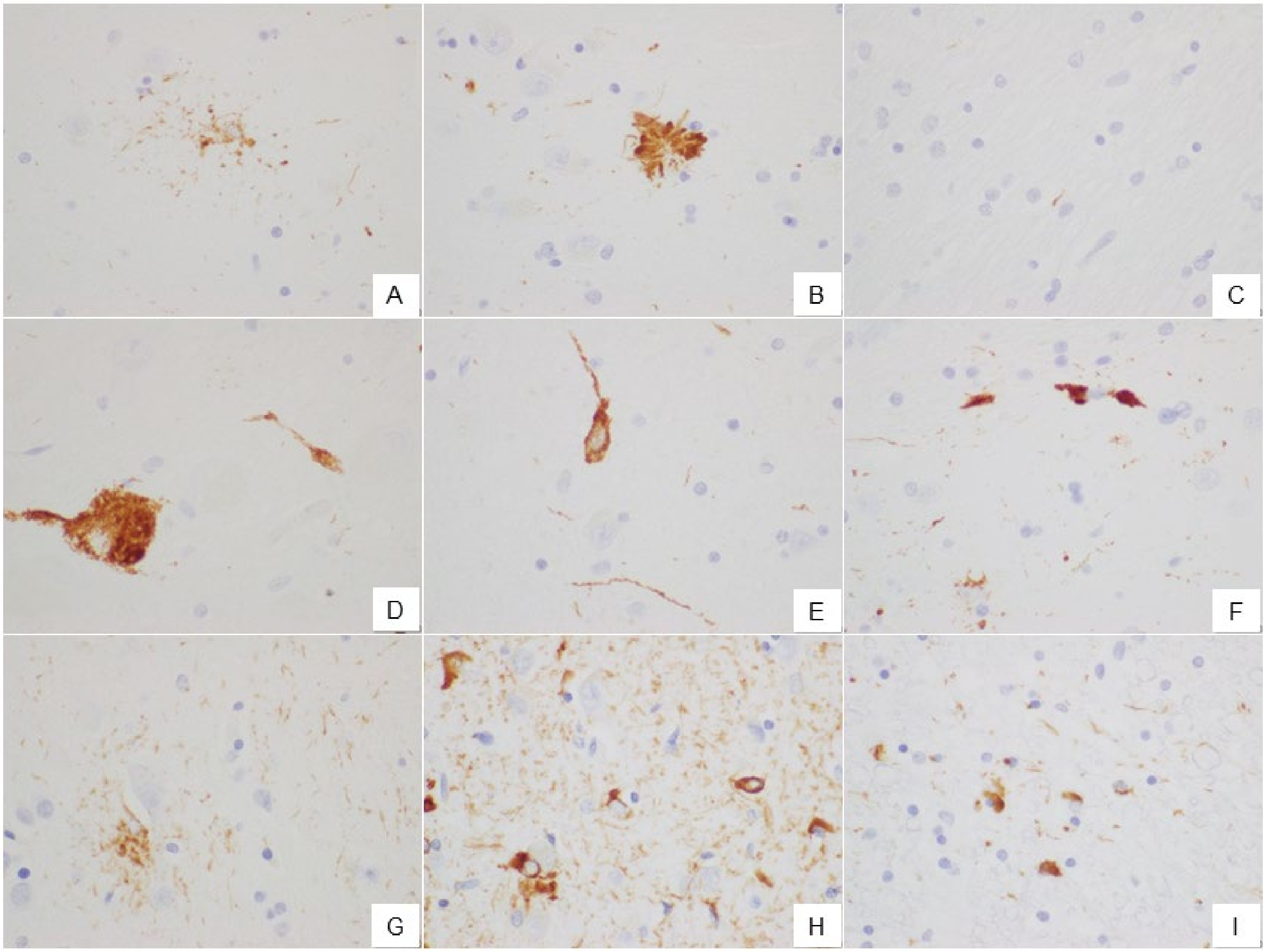

A. Corticomedial amygdala with unclassifiable astrocyte, B. basolateral amygdala with unclassifiable glial lesion, C. Anterior commisure with minimal threads but no globular glial inclusions. D. Basal nucleus with pretangles but minimal threads. E. Globus pallidus with pretangles and mild threads. F. Putamen with threads and glial lesions including globular glial inclusions. G-H. Precentral/postcentral cortices, G. tufted like astrocyte, H. globular astrocytic inclusions and coiled body like glial lesions. I. Underlying white matter with globular glial lesions and threads.

Figure 10

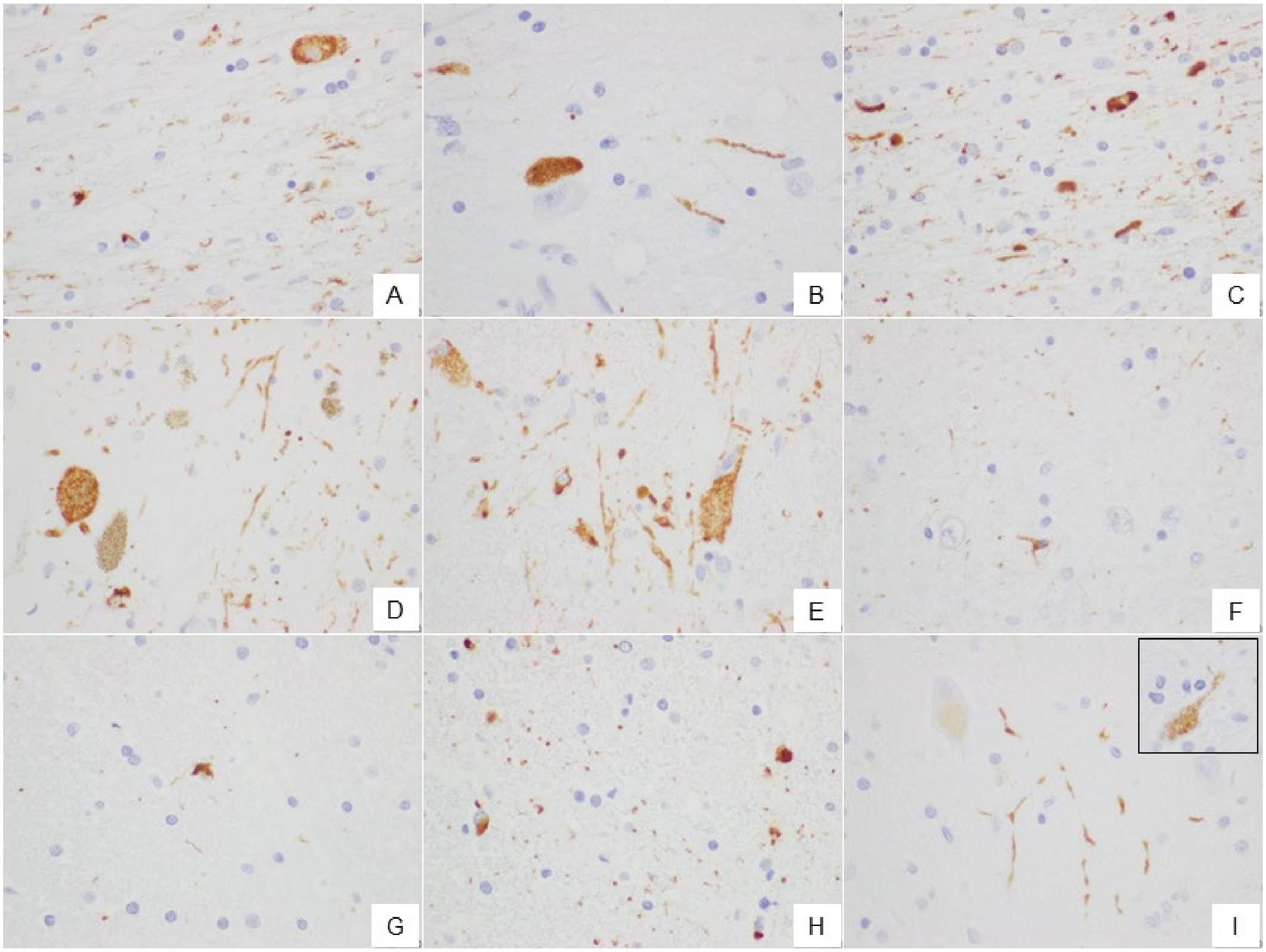

A. Subthalamic nucleus with threads, pretangles and glial lesions. B. Thalamus with pretangles and threads. C. Thalamic fasciculus with threads and globular glial inclusions. D. Substantia nigra with pretangles and threads. E. Red nucleus with pretangles, threads and glial lesions. F. Superior colliculus with mild thread and glial lesion. G. Cerebral peduncle, frontobulbar tract with isolated glial lesion. H. Cerebral peduncle, corticospinal tract with threads and glial lesion. I. Dentate nucleus with sparse threads and pretangle (inset).

Figure 11

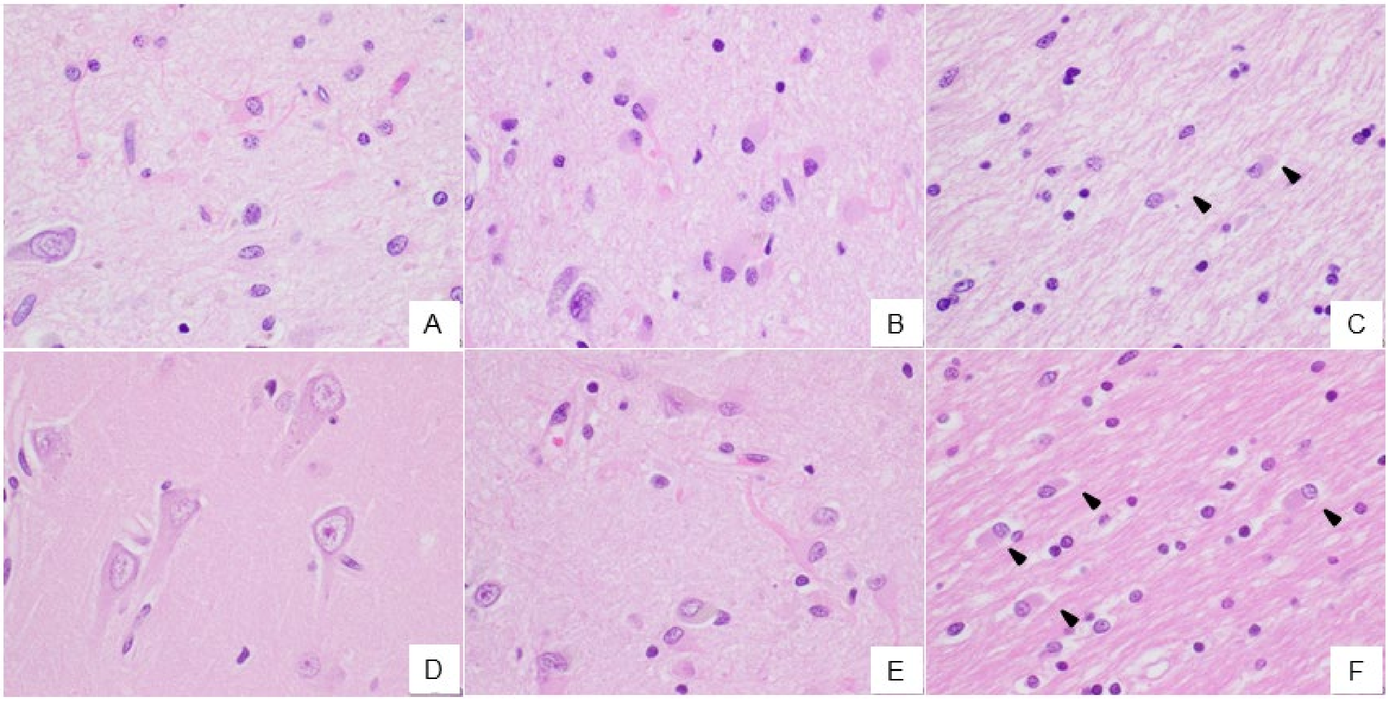

A-C. GGT type 1. A. Subiculum with severe neuronal loss and reactive astrogliosis. B. Corticomedial amygdala with severe neuronal loss and astrogliosis. C. Anterior commissure with severe myelin pallor. Arrowheads show globular glial inclusions. **D-F. GGT type 3. D**. Subiculum shows relative preservation. Neurons show granulovacuolar changes. E. Corticomedial amygdala with neuronal loss and reactive astrogliosis. F. Relative myelin preservation. Arrowheads show globular glial inclusions.

Globular Glial Tauopathies-GGT Type 1

Figure 12

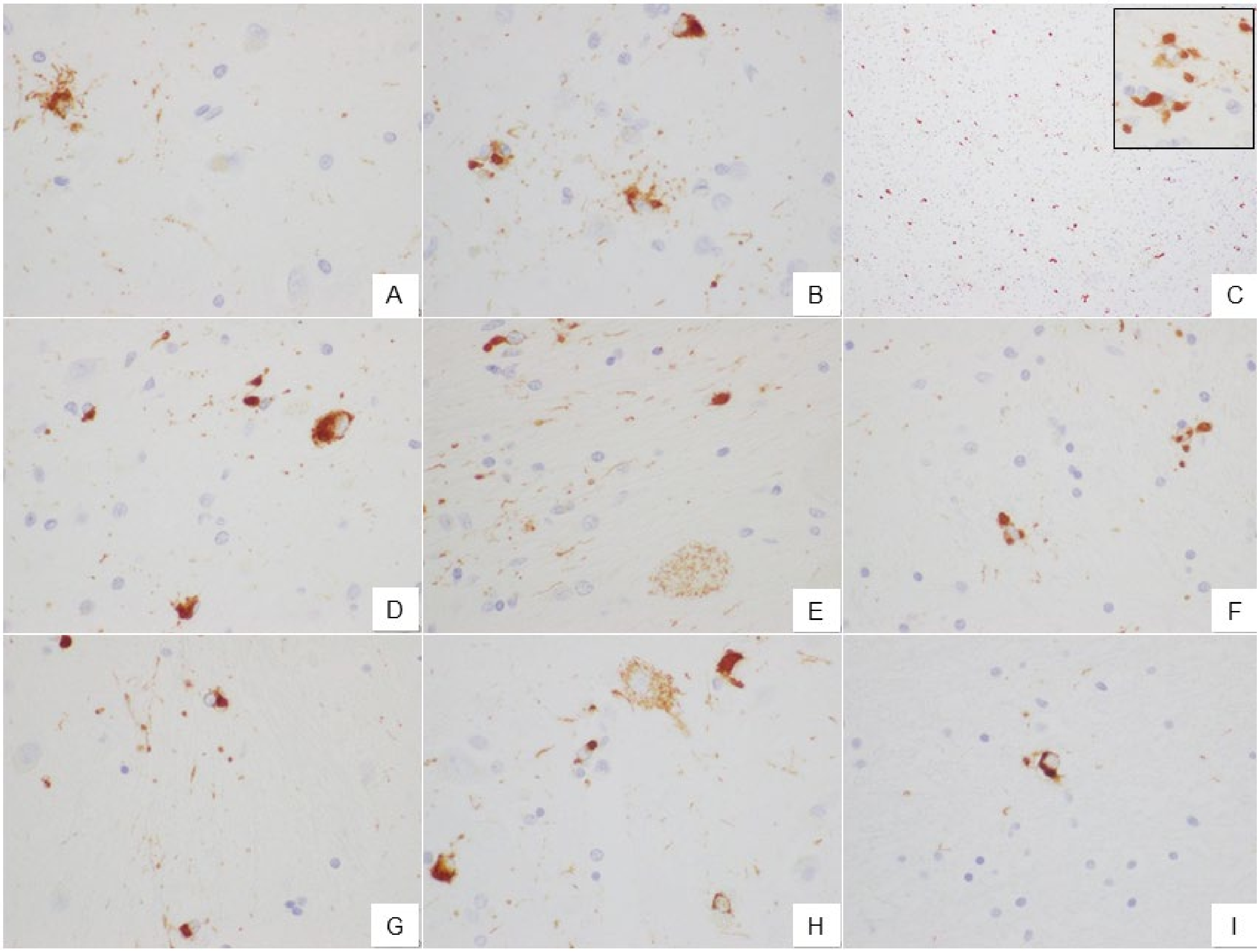

A. Subiculum, B. Entorhinal. C. Medial temporal white matter (x10), inset (x60). D. Amygdala with globular oligodendroglial inclusions. E. Basal nucleus of Meynert with pretangle, threads and globular oligodendroglial inclusions. F. Globus pallidus with sparse glial inclusions. G. Putamen with globular oligodendroglial inclusions, mild thread pathology. H. Motor cortex with pretangles and few globular glial inclusions. I. The subjacent motor white matter only shows mild pathology with isolated globular glial inclusions.

Figure 13

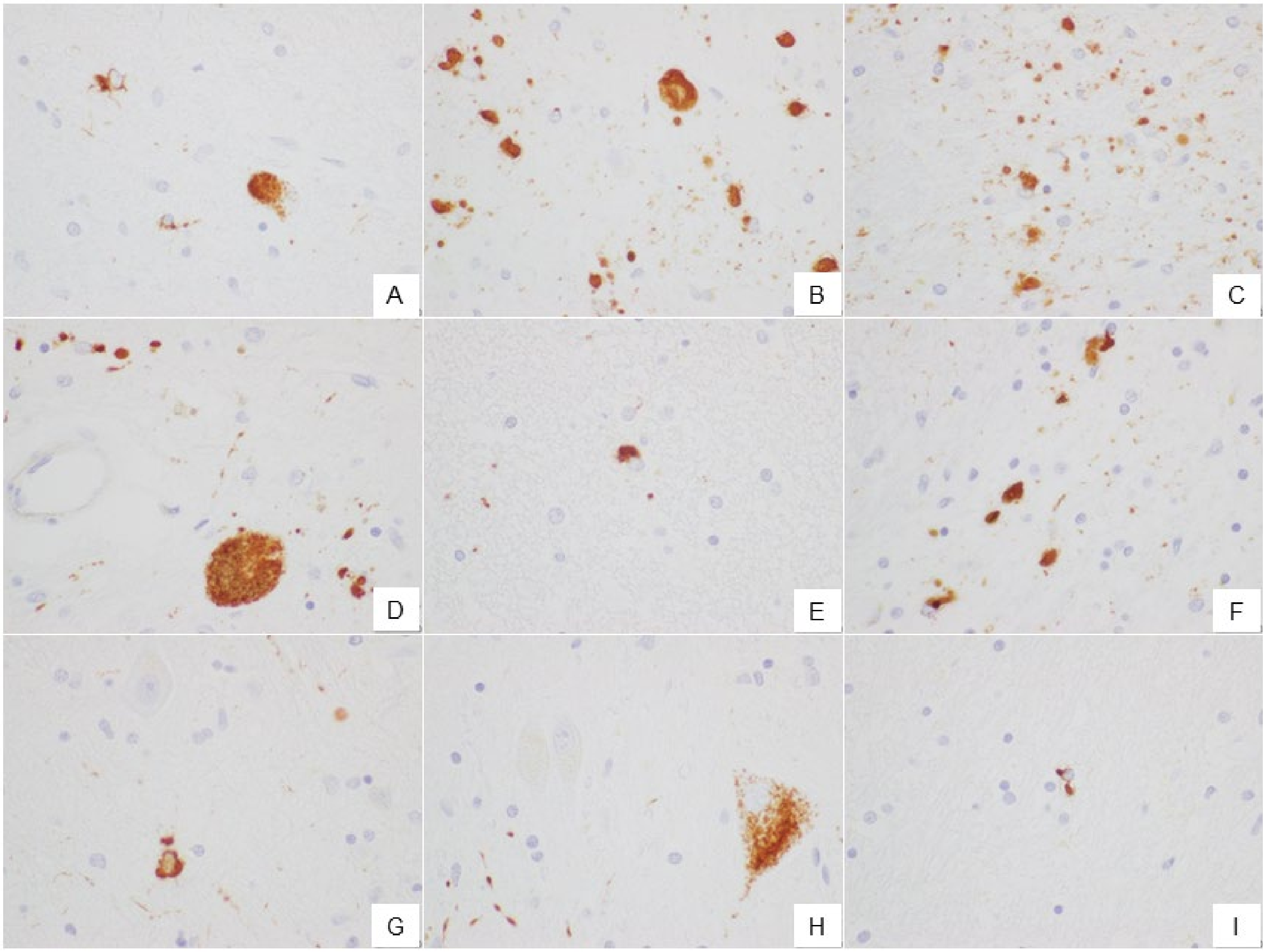

A. Subthalamic nucleus with mild pathology consisting of pretangles and glial lesions. B. Thalamus with pretangles and tangles and many globular glial inclusions. C. Thalamic fasciculus with many globular glial inclusions and thread pathology. D. Substantia nigra with pretangles and tangles and glial lesions. E. Gradient affection of the cerebral peduncle shows only mild affection of the corticospinal tract in contrast to F. frontobulbar tract with many glial inclusions. G. The superior colliculus shows only mild pathology. H. The cerebellar dentate nucleus showing pretangle and mild threads. I. The cerebellar white matter with only isolated glial lesions.

Globular Glial Tauopathies-GGT Type 3

Figure 14

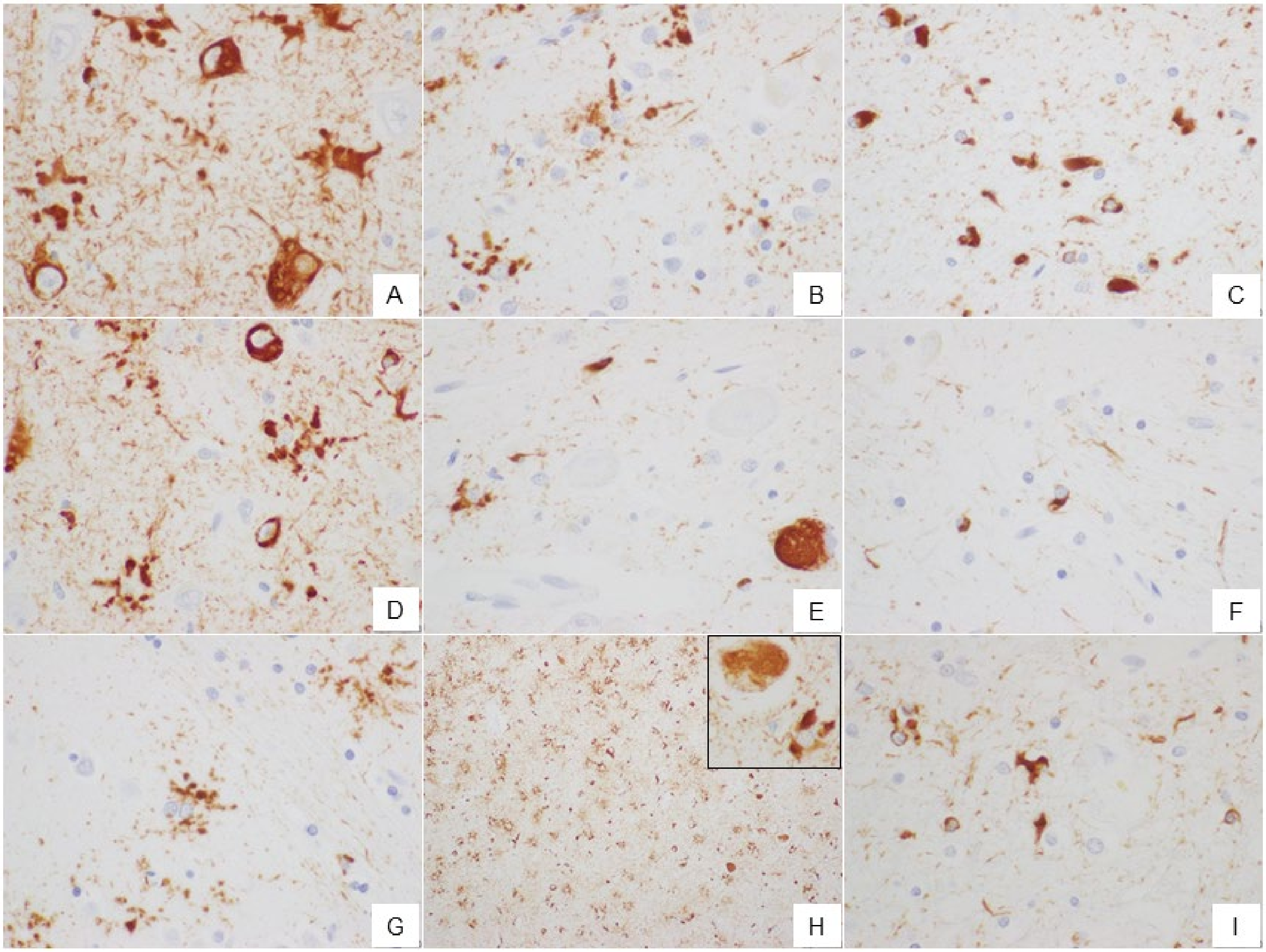

A. Subiculum with tangles and globular astrocytic inclusions. B. Entorhinal cortex with globular astrocytic inclusions. C. Medial temporal white matter with threads and globular oligodendroglial inclusions. D. Amygdala. E. Basal nucleus of Meynert. F. Globus pallidus with coiled bodies and threads. G. Putamen with globular astrocytic inclusions. H. Motor cortex marked globular astrocytic inclusions (x10), inset; tangles and globular astrocytic inclusions (x60). I. Subjacent motor white matter with threads and glial inclusions.

Figure 15

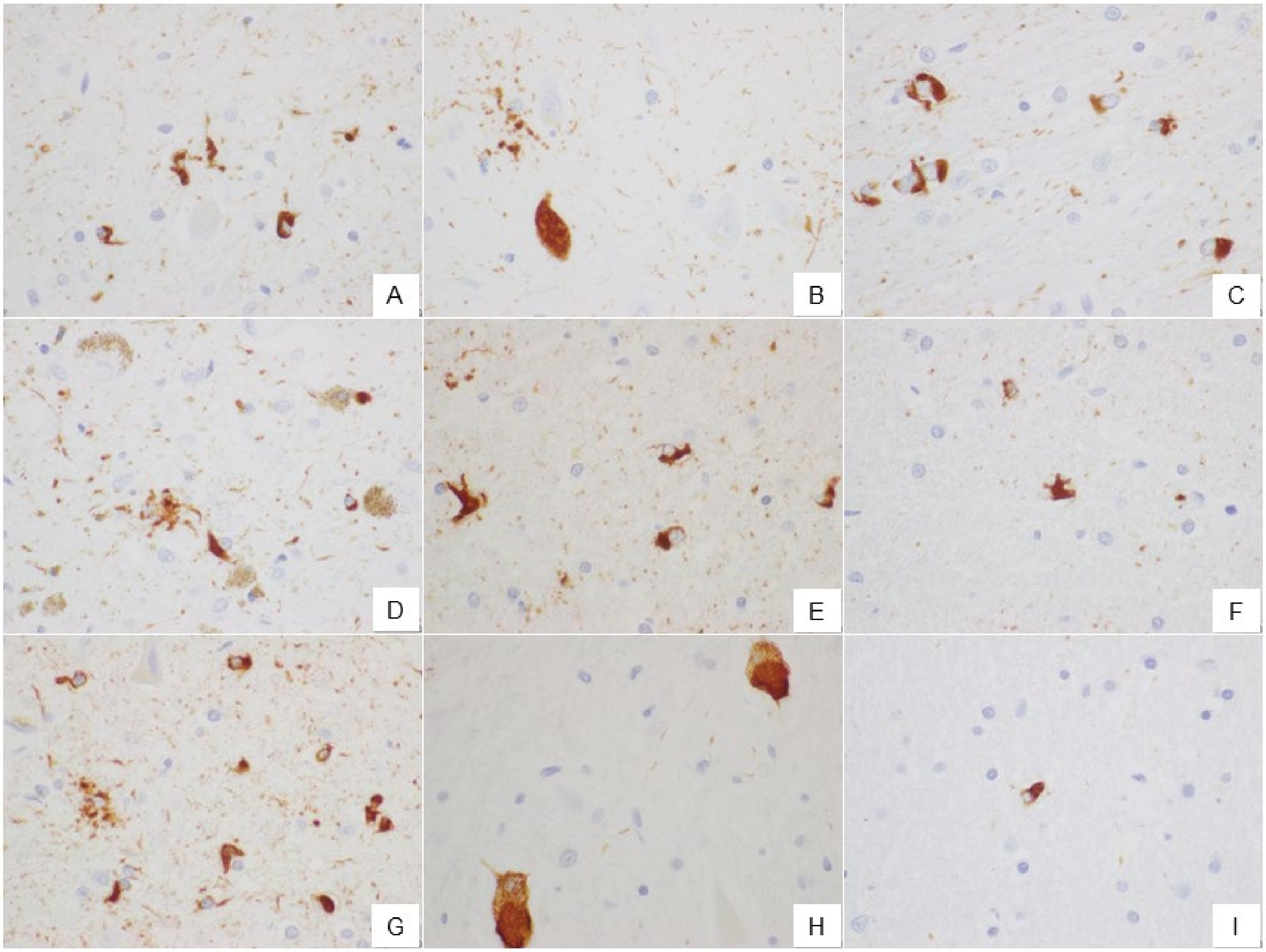

A. Subthalamic with threads and globular glial inclusions. B. Thalamus with tangle and astrocytic lesion. C. Thalamic fasciculus with threads and globular oligodendroglial inclusions. D. Substantia nigra with neuronal loss, astrocytic and oligodendroglial lesions. E. Cerebral peduncle, corticospinal tract with threads and many globular glial inclusions, contrasting F. frontobulbar tract which is less severely affected. G. Superior colliculus showing threads and many globular glial lesions. H. Dentate nucleus showing pretangles. I. Minimal affection of the cerebellar white matter.

Figure 16

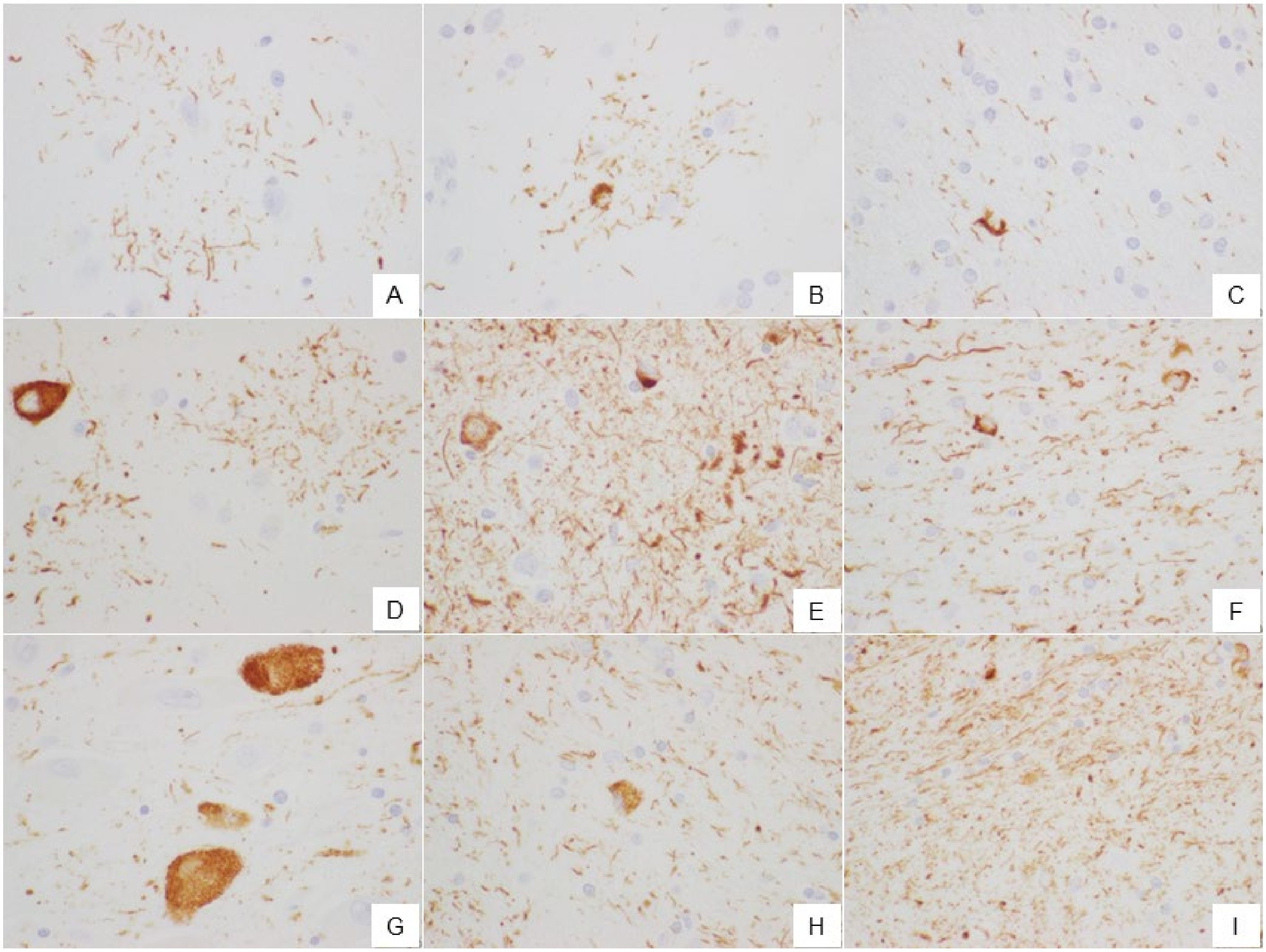

A. Subiculum with astrocytic plaque. B. Entorhinal cortex with pretangle and astrocytic plaque. C. Medial temporal white matter with threads and coiled body. D. Basolateral amygdala with tangle and astrocytic plaques. E. Precentral/postcentral cortices with pretangles marked threads. F. Subjacent white matter with threads and coiled bodies. G. Basal nucleus with pretangles and threads. H. Globus pallidus with threads and pretangles. I. Putamen with marked threads including the pencil fibers.

Corticobasal degeneration

Figure 17

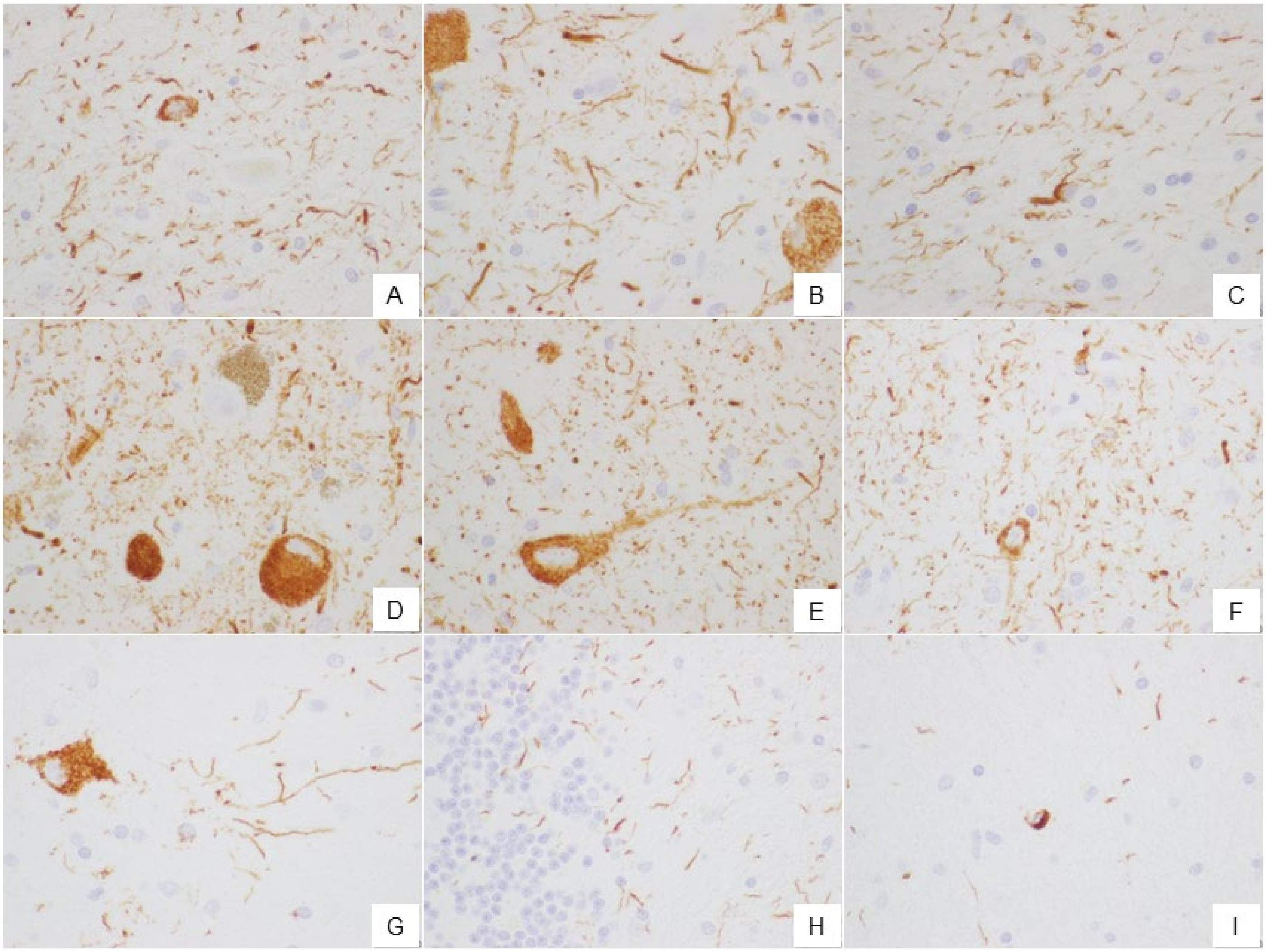

A. Subthalamic nucleus with threads and a coiled body. B. Thalamus with threads and pretangles. C. Thalamic fasciculus with threads and a coiled body. D. Substantia nigra with threads and pretangles and tangles. E. Red nucleus with threads and pretangles. F. Superior colliculus with threads and a pretangle. G. Dentate nucleus with pretangle and threads. H. Cerebellar white matter with threads. I. cerebellar white matter showing mild threads and isolated coiled bodies.

Argyrophilic Grain Disease-diffuse

Figure 18

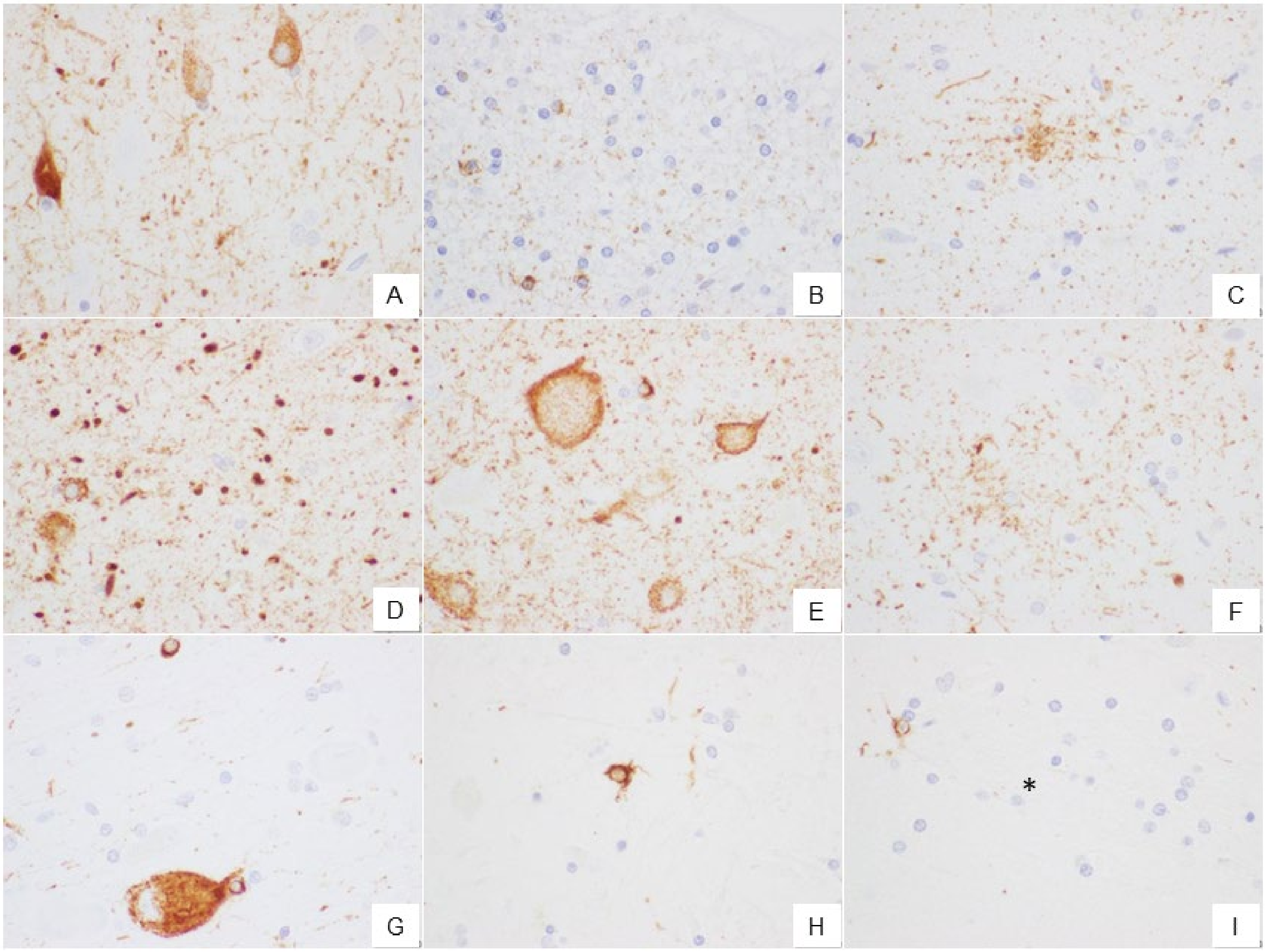

A. Subiculum with threads, pretangles and grains. B. Fornix with threads and coiled bodies. C. Entorhinal cortex with threads and unclassifiable glial lesion. D-F. Amygdala. D. Grains and pretangles. E. Ballooned neurons, threads and grains. F. Basolateral amygdala with fuzzy but not so granular astrocyte. G. Basal nucleus with pretangle and coiled bodies. H. Globus pallidus with sparse threads and a coiled body. I. Putamen with an isolated coiled body. * Pencil fibers are spared.

Pick's Disease

Figure 19

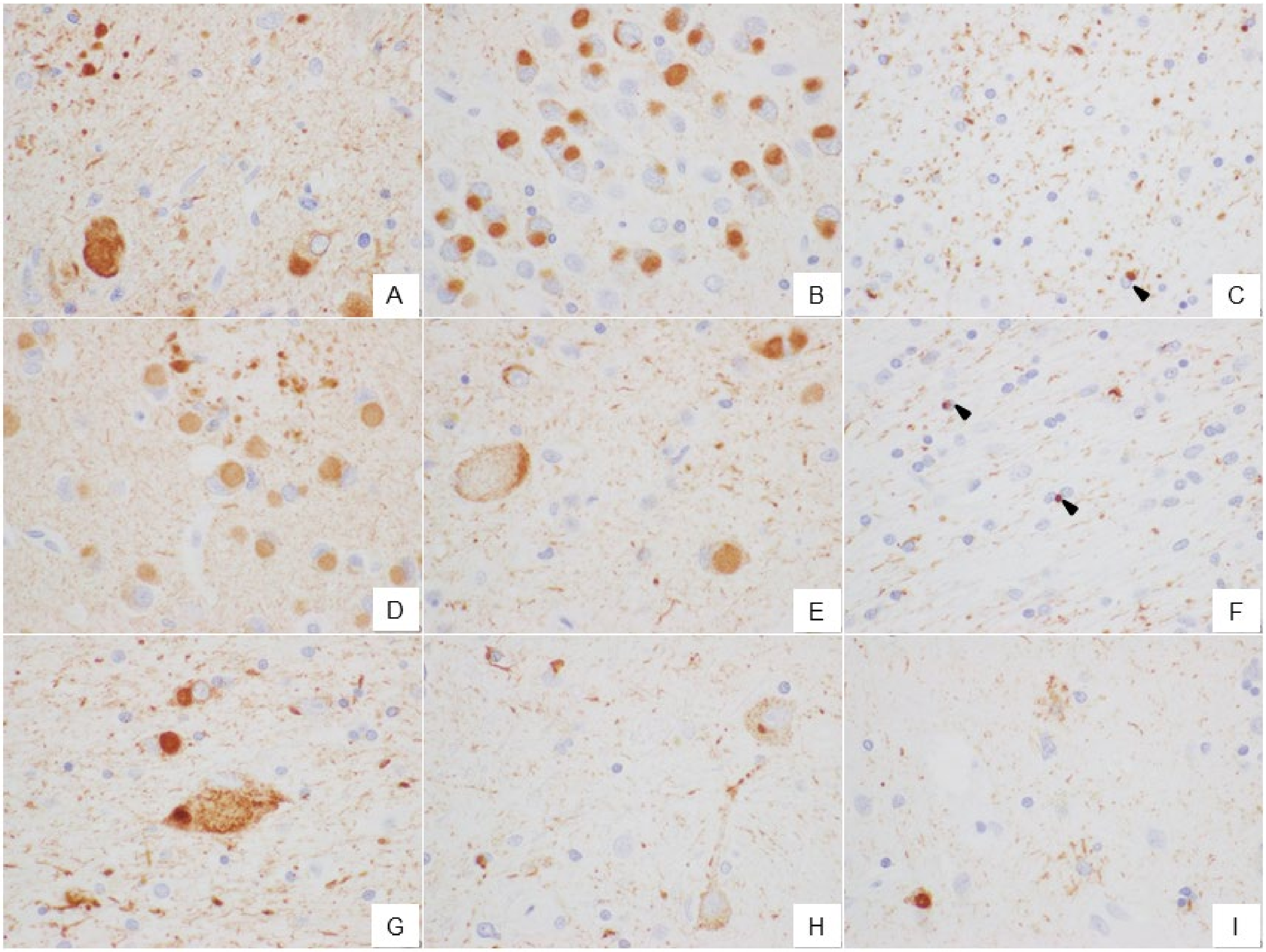

A. Subiculum with tangle, Pick body and threads. B. Dentate fascia with many Pick bodies. C. Medial temporal white matter with marked thread and small globular inclusion (arrowhead). D-E. Amygdala shows Pick bodies and a neuritic plaque. E. Ballooned neuron and Pick bodies. F. Anterior commissure with moderate threads and small globular glial inclusions (arrowheads). Basal nucleus with Pick bodies. H. Globus pallidus with coiled body, threads and pretangles. I. Putamen with astroglial lesions and a Pick body.

Figure 20

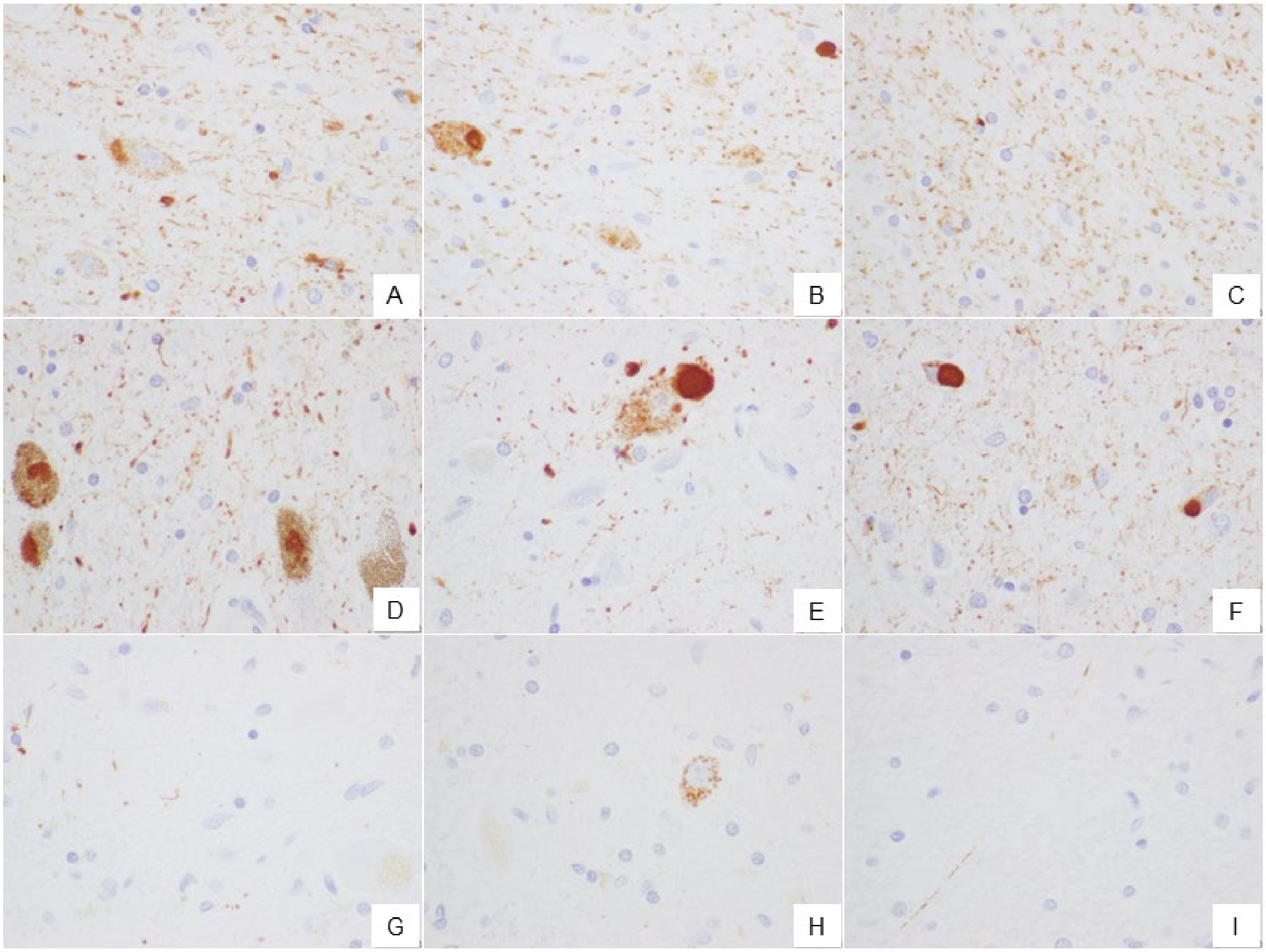

A. Subthalamic nucleus with threads, a pretangle and glial tau. B. Thalamus with pretangles, Pick bodies and threads. C. Thalamic fasciculus with thread and a small globular inclusion. D. Substantia nigra with neuronal inclusions and threads. E. Red nucleus with threads, pretangle and Pick body. F. Superior colliculus with threads and Pick bodies. G-H. Dentate nucleus. G. glial lesion and H. pretangle. I. Cerebellar white matter with sparse threads.

Figure 21

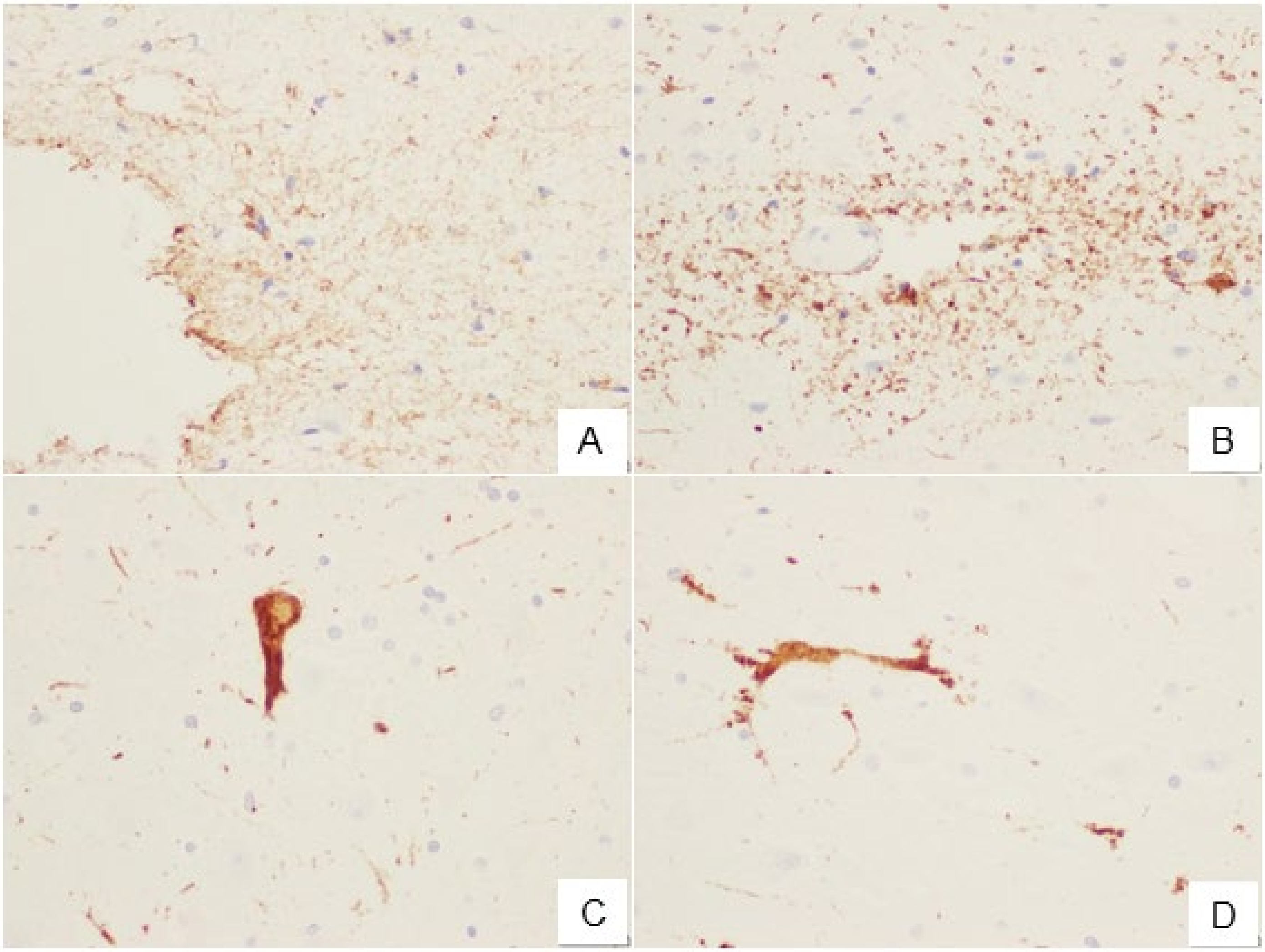

A. ARTAG like pathology in the depth of the cortical sulcus. **B.** Patchy perivascular tau and **C.** tangles. **D.** Endplate with dendritic neuritic dystrophy.

Figure 22

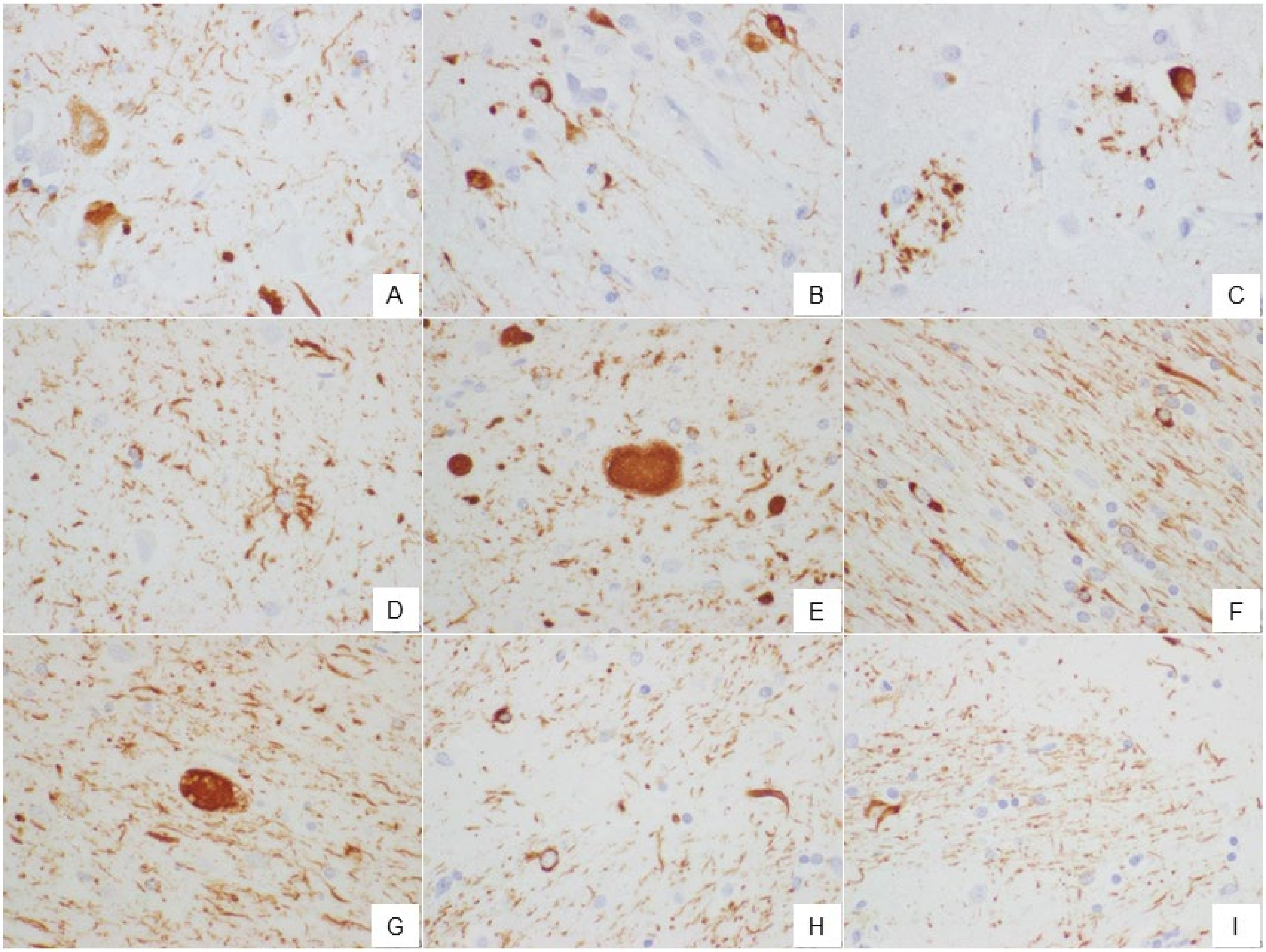

A. Subiculum with threads, pretangles and grains. B. Dentate fascia with pretangles and tau positive apical dendrites. C. Entorhinal cortex showing unclassified plaque like glial lesions and a ballooned neuron. D. Corticomedial amygdala with threads and unclassified glial lesion. E. Basolateral amygdala with enlarged 'grains', threads and a tangle. F. Anterior commissure with marked threads and oligodendroglial globular inclusions. G. Basal nucleus of Meynert with threads and a tangle. H. globus pallidus with threads and coiled body like glial lesions. I. Putamen with marked thread pathology and coiled body like glial lesion.

Figure 23

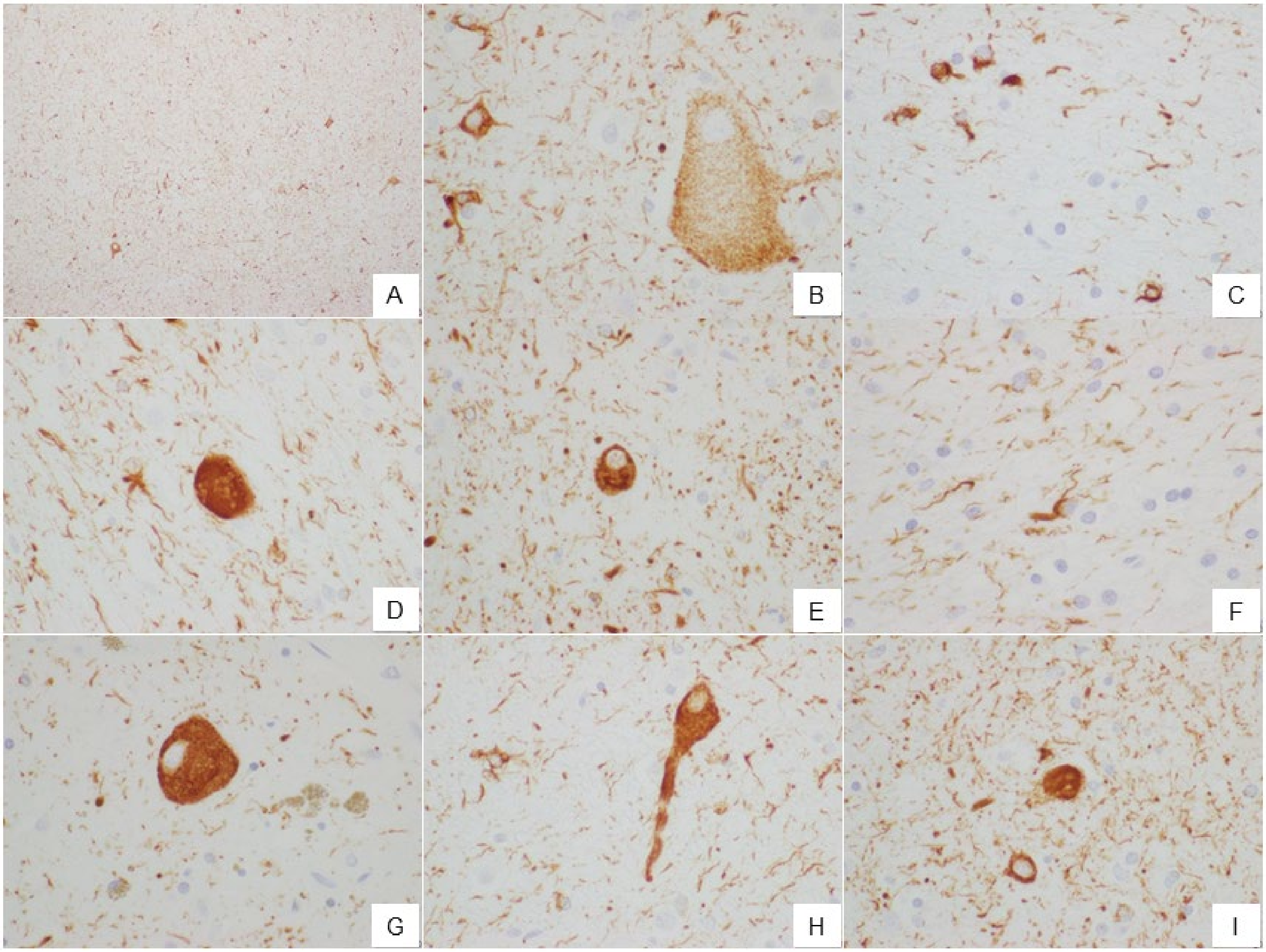

A. Motor cortex with marked thread pathology. B. Higher power showing threads, coiled body like inclusions, and a ballooned neuron. C. Subjacent motor white matter with threads and coiled bodies. D. Subthalamic nucleus with threads and a tangle. E. Thalamus with threads, grains, and a pretangle. F. Thalamic fasciculus with threads and coiled body like glial lesion. G. Substantia nigra with threads, grains and a tangle. H. Red nucleus with threads, coiled body like glial pathology and a pretangle. I. Superior colliculus with marked threads and pretangles (vs coiled body like inclusion).

Block key

Region	Block
Middle frontal cortex	A
Precentral/postcentral gyri	D
Occipital cortex	E
Hippocampus	F
Amygdala	G
Globus pallidus	G
Putamen	G
Subthalamic nucleus	K
Substantia Nigra	M
Dentate nucleus	P

Required PSP lesions

Table 1. Minimal PSP Criteria (yes/no PSP)				
Region	Tau lesion			
	Pretangles/ tangles		Tufted astrocytes	
	NO	YES	NO	YES
Motor				
Globus Pallidus				
Putamen				
Subthalamic				
Substantia nigra				

Tauopathy Scoring Using Online Qualtrics

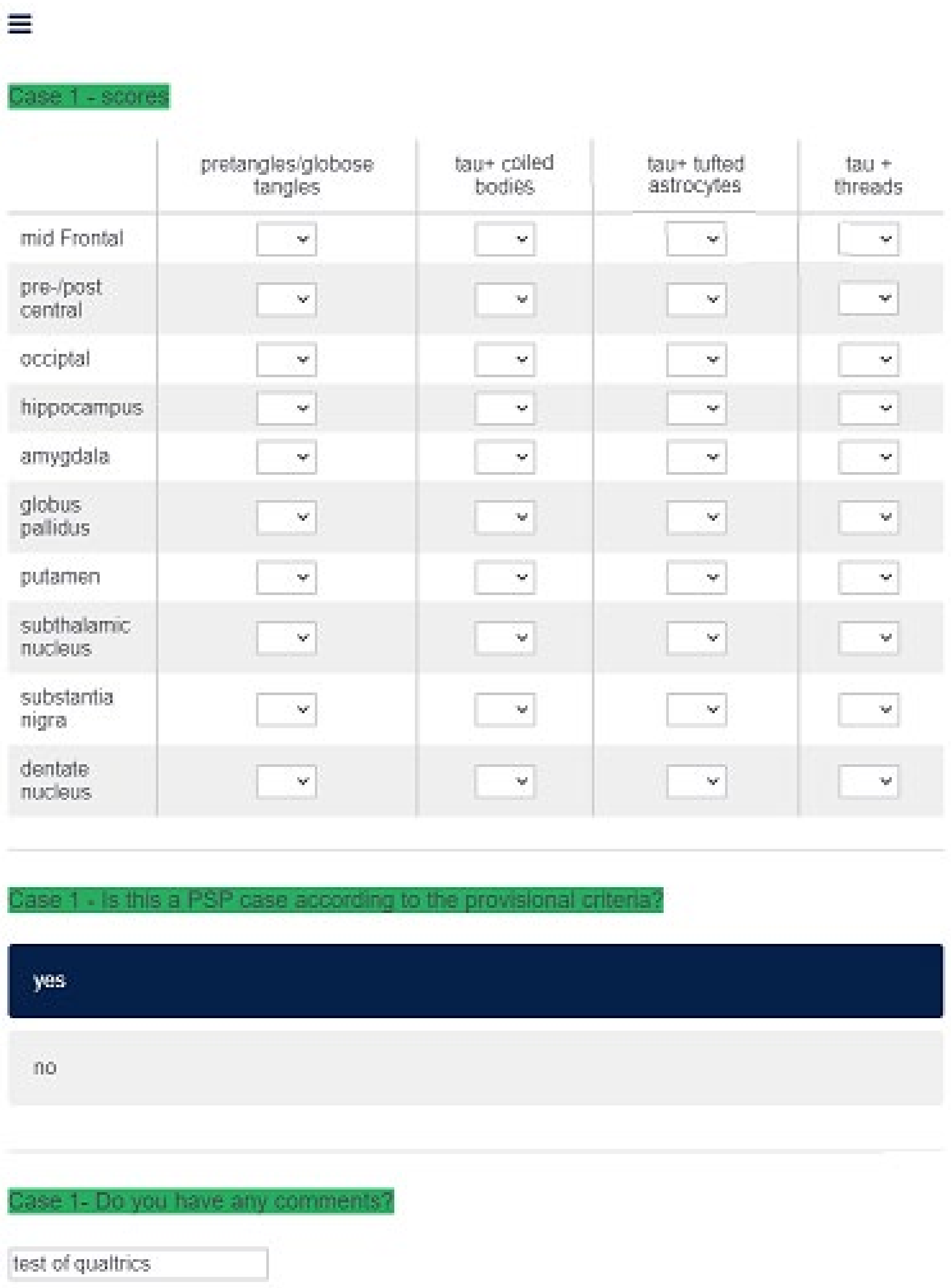

Case 1 - scores

	pretangles/globose tangles	tau+ coiled bodies	tau+ tufted astrocytes	tau + threads
mid Frontal				
pre-/post central				
occipital				
hippocampus				
amygdala				
globus pallidus				
putamen				
subthalamic nucleus				
substantia nigra				
dentate nucleus				

Case 1 - Is this a PSP case according to the provisional criteria?

yes

no

Case 1- Do you have any comments?

test of qualtrics

Summary of provisional neuropathologic diagnostic criteria of PSP using commonly affected anatomical regions included in the study.

2.1 The PSP cardinal nuclei (median scores)

	Tangles & pretangles	Threads	Astrocytes (tufted)	Oligodendroglia (coiled bodies)
**Substantia nigra**	2	2	1	1
**Subthalamic**	3	3	2	2
**Globus pallidus**	2	2	1	2

Cardinal nuclei are affected in both typical PSP and its variants.

2.2 Associated regions commonly affected (median scores)

	Tangles & pretangles	Threads	Astrocytes (tufted)	Oligodendroglia (coiled bodies)
**Motor cortex**	2	2	3	2
**Putamen**	2	1	3	2
**Dentate nucleus**	2	2	0	1

The morphology of tufted astrocytes may be less conspicuous in PSP cases that stand out either due to mild ('early') pathology or atypical variants. In these cases, cardinal nuclei are similarly affected with tau lesions as in typical PSP, but tau positive astrocytes may show atypical morphology.

Contact Information

For technical issues related to the use of the Aperio server please email:

Kristen Whitney
Icahn Medical School at Mount Sinai, New York
kristen.whitney@mssm.edu

For general questions or concerns please contact:

Rachel R. LaPaille-Harwood
Brain bank coordinator, Mayo Clinic
Department of Neuroscience Research
Jacksonville, FL
Phone: 904-953-2439
Fax: 904-953-7117
Email: Lapaille-Harwood.rachel@mayo.edu

Laura Wise
Administrative Officer
Weill Institute for Neuroscience
Memory and Aging Center
University of California, San Francisco, CA
Phone: 415-514-8953
Email: Laura.Wise@ucsf.edu
