# Supplementary material for: Rainwater Charitable Foundation criteria for the neuropathologic diagnosis of progressive supranuclear palsy
Source: Acta Neuropathol. 2022 Aug 10;144(4):603–14. doi: 10.1007/s00401-022-02479-4 (PMC9468104; doi:10.1007/s00401-022-02479-4)
Supplement: Supplementary file 2 — Supplementary file2 (DOCX 10272 kb) [file 401_2022_2479_MOESM2_ESM.docx]

**Supplementary Figure 2**


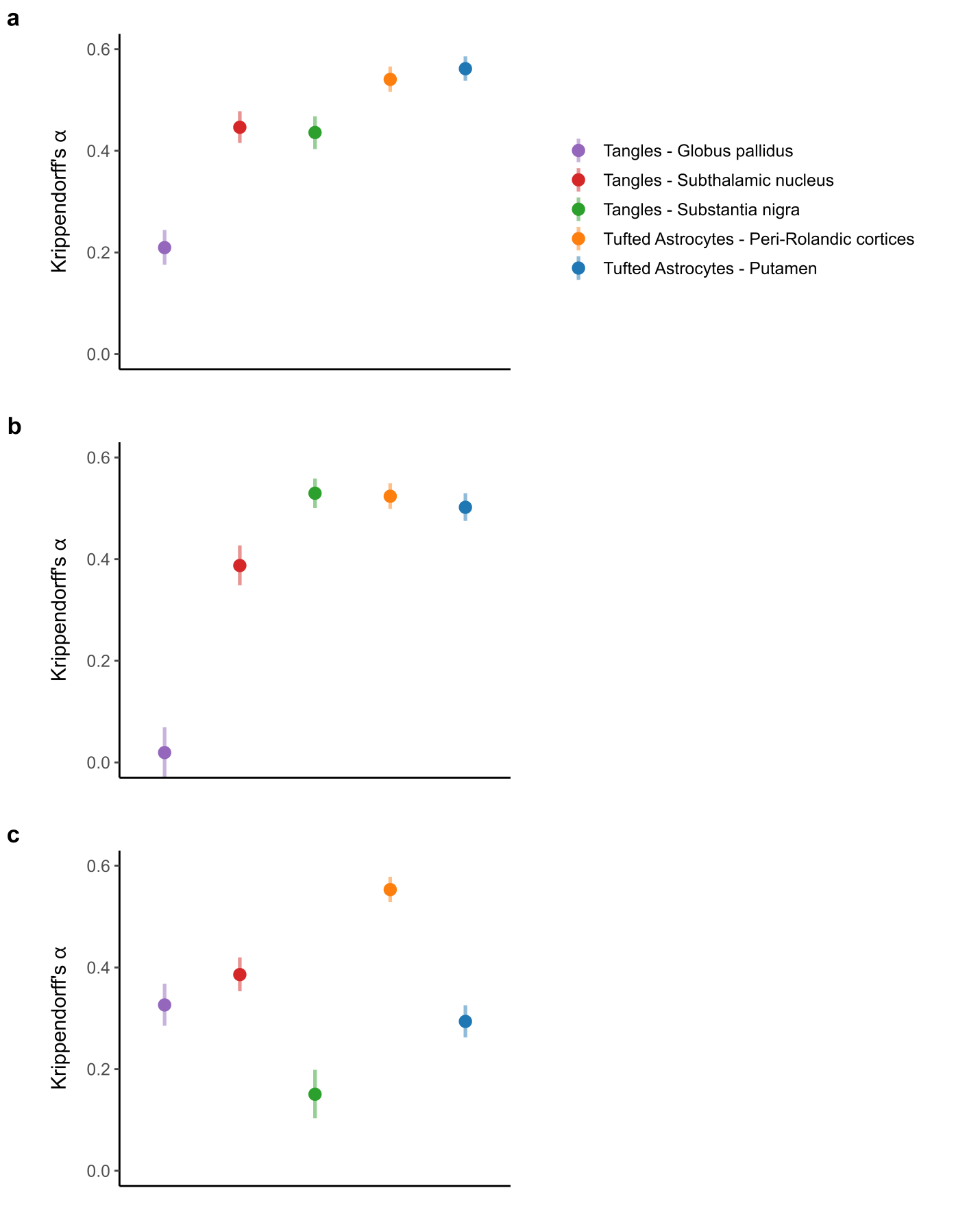


Semiquantitative scores from each rater using their personal preference expectedly show a wide variation in individual scores. In the all 15 cases of PSP (a), and in typical PSP (b) tufted astrocytes in the peri-Rolandic cortices and putamen show the best inter-rater reliability. Neurofibrillary tangles in the globus pallidus show the lowest inter-rater reliability. Atypical PSP cases (c) show the highest inter-rater reliability in the peri-Rolandic cortex and the lowest inter-rater reliability for neurofibrillary tangles in the substantia nigra.

**Supplementary Figure 3**

**
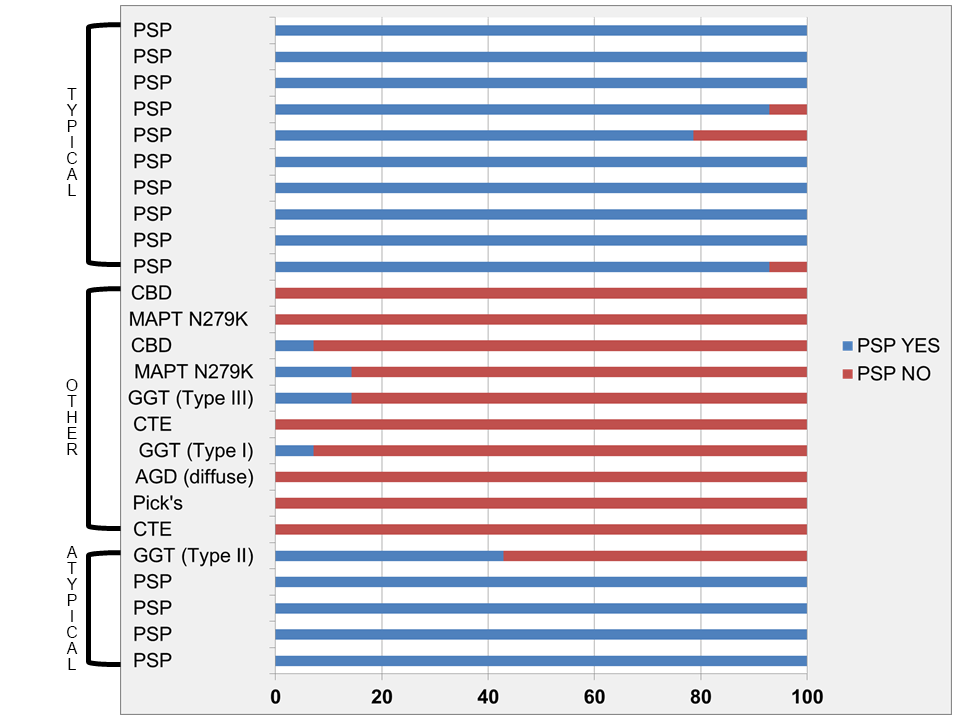
**

The bar graph shows the percentage of inter-rater agreement per case with the breakdown of the Mayo Clinic diagnoses. For easy readability, the cases are shown per subgroup; typical (typical PSP), other (other tauopathy), atypical (atypical PSP), and not in the order of how the cases were presented to the raters (#1-25). The blue color denotes the percentage of raters who assigned a diagnosis of PSP. The red color represents the percentage of raters who assigned a diagnosis of not-PSP.

**Supplementary Figure 4**

**
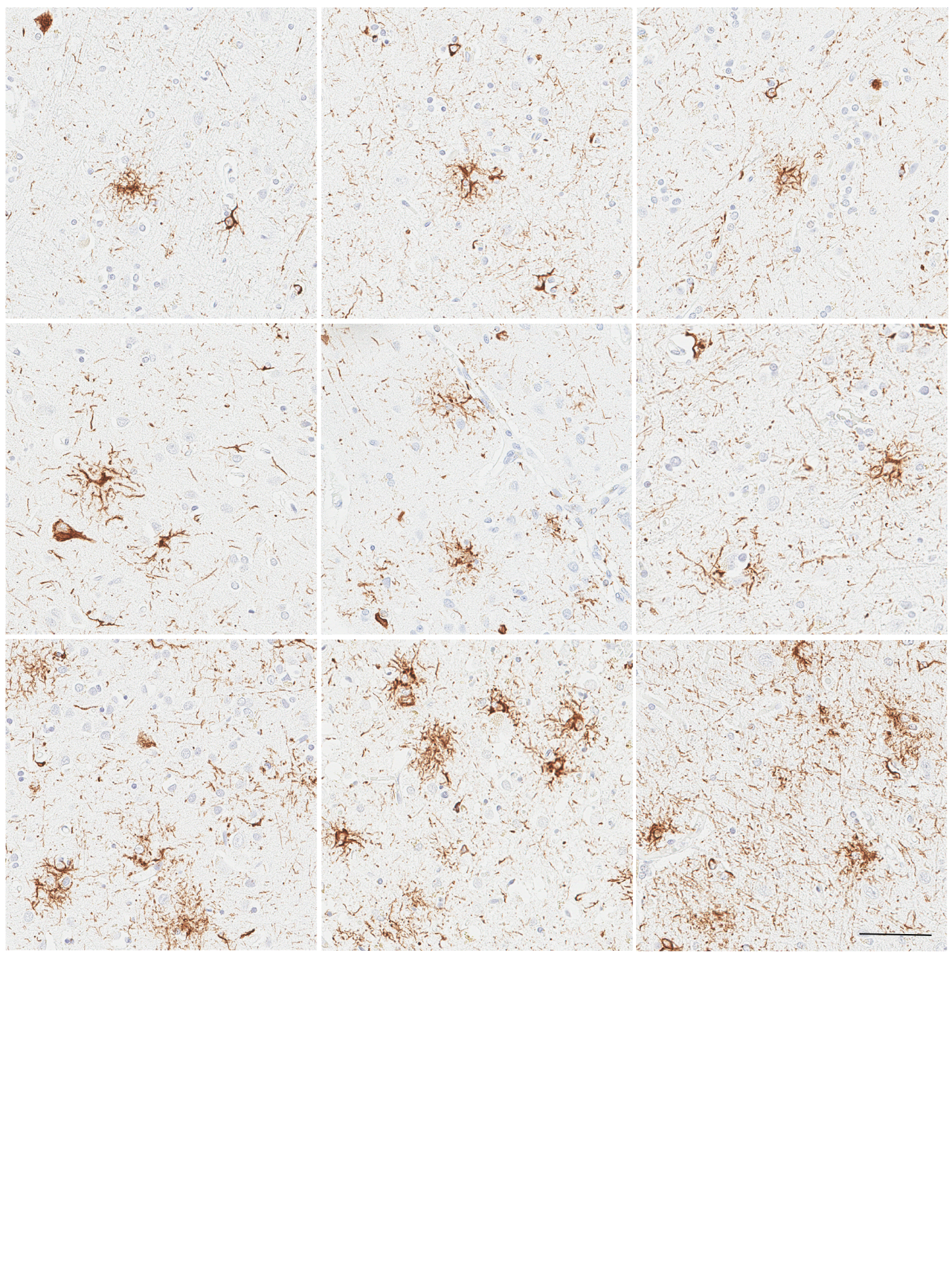
**

Inter-rater agreement in PSP cases was high, with >70% of PSP cases showing a complete agreement. The images represent some cases with complete agreement, showing a range in tufted astrocyte burden and morphology. All represented images are from PSP patients. The captured images are from digitally scanned slides of the primary motor cortex (peri-Rolandic motor strip) stained for phosphorylated tau (AT8). Scale bar = 60 µm.

**Supplementary Figure 5**

**
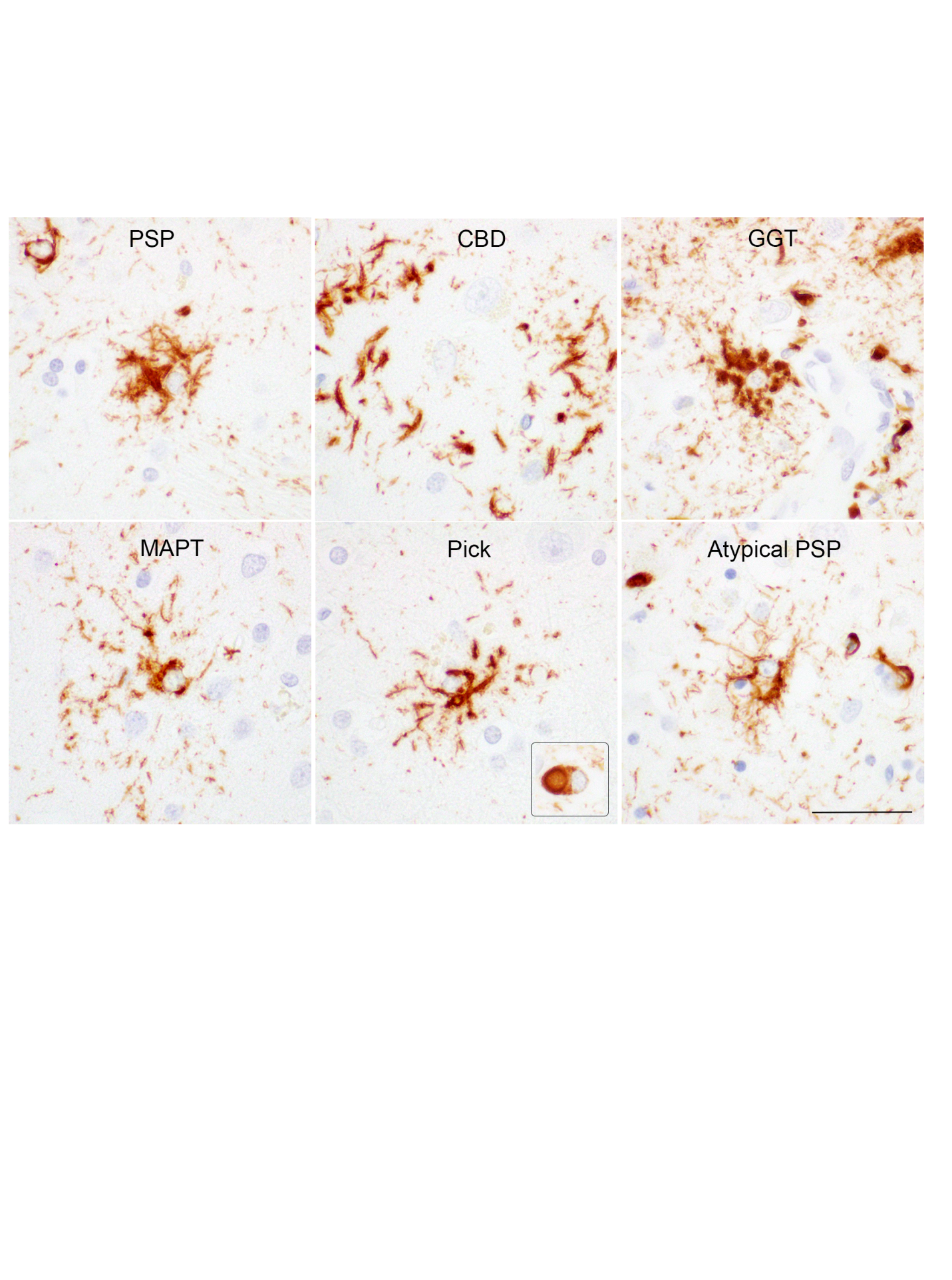
**

Representative images of astrocytic tau positive lesions in PSP and other tauopathies showing tufted astrocyte in typical PSP, an astrocytic plaque in CBD, an astrocyte with globular glial inclusions in GGT, an unclassified tau positive astrocyte in a mutation carrier (N279K), a ramified astrocyte in Pick’s disease (inset shows a neuronal Pick body) and a tufted-like astrocyte in atypical PSP. All images were stained for phosphorylated tau (AT8) and captured at 60x magnification on an Olympus BX41 microscope, using digital the camera DP22. Scale bar = 20 µm.
